# Gene replacement therapy in a model of Charcot-Marie-Tooth 4C neuropathy

**DOI:** 10.1093/brain/awz064

**Published:** 2019-03-25

**Authors:** Natasa Schiza, Elena Georgiou, Alexia Kagiava, Jean-Jacques Médard, Jan Richter, Christina Tryfonos, Irene Sargiannidou, Amanda J Heslegrave, Alexander M Rossor, Henrik Zetterberg, Mary M Reilly, Christina Christodoulou, Roman Chrast, Kleopas A Kleopa

**Affiliations:** 1 Neuroscience Laboratory and Neurology Clinics, The Cyprus Institute of Neurology and Genetics and Cyprus School of Molecular Medicine, Nicosia, Cyprus; 2 Department of Neuroscience and Department of Clinical Neuroscience, Karolinska Institutet, Stockholm, Sweden; 3 Department of Molecular Virology, The Cyprus Institute of Neurology and Genetics and Cyprus School of Molecular Medicine, Nicosia, Cyprus; 4 Department of Neurodegenerative Disease, UCL Institute of Neurology, London, UK; 5 UK Dementia Research Institute at UCL, London, UK; 6 Department of Neuromuscular Diseases, UCL Institute of Neurology and National Hospital for Neurology and Neurosurgery, London, UK; 7 Department of Psychiatry and Neurochemistry, Institute of Neuroscience and Physiology, the Sahlgrenska Academy at the University of Gothenburg, Mölndal, Sweden; 8 Clinical Neurochemistry Laboratory, Sahlgrenska University Hospital, Mölndal, Sweden

**Keywords:** Charcot-Marie-Tooth 4C disease, peripheral neuropathy, gene therapy, Schwann cells, biomarkers

## Abstract

Charcot-Marie-Tooth disease type 4C is the most common recessively inherited demyelinating neuropathy that results from loss of function mutations in the *SH3TC2 *gene. *Sh3tc2*^−/−^ mice represent a well characterized disease model developing early onset progressive peripheral neuropathy with hypo- and demyelination, slowing of nerve conduction velocities and disturbed nodal architecture. The aim of this project was to develop a gene replacement therapy for treating Charcot-Marie-Tooth disease type 4C to rescue the phenotype of the *Sh3tc2*^−/−^ mouse model. We generated a lentiviral vector LV*-Mpz.SH3TC2.myc* to drive expression of the human *SH3TC2* cDNA under the control of the *Mpz* promoter specifically in myelinating Schwann cells. The vector was delivered into 3-week-old *Sh3tc2*^−/−^ mice by lumbar intrathecal injection and gene expression was assessed 4–8 weeks after injection. Immunofluorescence analysis showed presence of myc-tagged human SH3TC2 in sciatic nerves and lumbar roots in the perinuclear cytoplasm of a subset of Schwann cells, in a dotted pattern co-localizing with physiologically interacting protein Rab11. Quantitative PCR analysis confirmed *SH3TC2* mRNA expression in different peripheral nervous system tissues. A treatment trial was initiated in 3 weeks old randomized *Sh3tc2*^−/−^ littermate mice which received either the full or mock (LV-*Mpz.Egfp*) vector. Behavioural analysis 8 weeks after injection showed improved motor performance in rotarod and foot grip tests in treated *Sh3tc2*^−/−^ mice compared to mock vector-treated animals. Moreover, motor nerve conduction velocities were increased in treated *Sh3tc2*^−/−^ mice. On a structural level, morphological analysis revealed significant improvement in g-ratios, myelin thickness, and ratios of demyelinated fibres in lumbar roots and sciatic nerves of treated *Sh3tc2*^−/−^ mice. Finally, treated mice also showed improved nodal molecular architecture and reduction of blood neurofilament light levels, a clinically relevant biomarker for axonal injury/degeneration. This study provides a proof of principle for viral gene replacement therapy targeted to Schwann cells to treat Charcot-Marie-Tooth disease type 4C and potentially other similar demyelinating inherited neuropathies.

## Teaser

Charcot-Marie-Tooth type 4C is a demyelinating neuropathy caused by mutations of *SH3TC2.* Schiza *et al.* report that intrathecal injection of a lentiviral vector for targeted *SH3TC2* delivery into Schwann cells leads to functional and morphological improvements in a disease model. This study provides proof of principle for treating demyelinating neuropathies.

## Introduction

Charcot-Marie-Tooth disease type 4 (CMT4) includes several recessively inherited demyelinating neuropathy forms caused by mutations in different genes, which lead to loss of function of the encoded proteins. CMT4C appears to be the most prevalent among this overall rare group of neuropathies, accounting for almost half of all CMT4 cases (Fridman *et al.*, 2015). Patients with CMT4C usually present in the first decade of life with delayed walking, progressive distal muscle atrophy and weakness, areflexia and sensory loss. Almost all patients develop foot deformities and scoliosis, often requiring surgery (Kessali *et al.*, 1997; Gabreels-Festen *et al.*, 1999; Azzedine *et al.*, 2006). Cranial nerve involvement with severe hearing loss, slow pupillary light reflexes, and lingual fasciculations are common with phenotypic variations within families (Gooding *et al.*, 2005; Colomer *et al.*, 2006; Varley *et al.*, 2015). A combination of proprioceptive loss and vestibular neuropathy may cause disabling imbalance early in disease evolution (Perez-Garrigues *et al.*, 2014). Electrophysiological studies in CMT4C patients confirm the demyelinating process with mean median motor nerve conduction velocity (MNCV) of 22.6 m/s. Nerve biopsies were characterized by an increase of basement membranes around myelinated, demyelinated, and unmyelinated axons, relatively few onion bulbs, and, most typically, large cytoplasmic extensions of Schwann cells (Kessali *et al.*, 1997; Gabreels-Festen *et al.*, 1999; Senderek *et al.*, 2003).

Linkage analysis studies and homozygosity mapping (LeGuern *et al.*, 1996) identified the CMT4C disease locus on chromosome 5q32, with subsequent discovery of several mutations in the *SH3TC2*/KIAA1985 gene (Senderek *et al.*, 2003). At least 28 different *SH3TC2 *mutations have been described to date, mostly truncating but also missense, with higher frequency among certain ethnic groups (Lassuthova *et al.*, 2011) likely due to founder effects (Gooding *et al.*, 2005). The gene covers 62 kb of genomic sequence and consists of at least 18 variably spliced exons. *SH3TC2 *encodes a protein of 1288 amino acids containing two Src homology 3 (SH3) and 10 tetratricopeptide repeat (TPR) domains sharing no overall significant similarity to any other human protein with known function. The presence of SH3 and TPR domains suggests that SH3TC2 could act as a scaffold protein (Senderek *et al.*, 2003). *SH3TC2 *is well conserved among vertebrate species, whereas no non-vertebrate orthologues were identified. SH3TC2 is present in several components of the endocytic pathway including early and late endosomes, and clathrin-coated vesicles close to the trans-Golgi network and in the plasma membrane. This localization is altered in CMT4C mutants examined *in vitro* (Lupo *et al.*, 2009).

A mouse model of CMT4C has been generated and studied in detail by replacing exon 1 of the *Sh3tc2 *gene with an enhanced GFP (eGFP)-Neo cassette (Arnaud *et al.*, 2009). *Sh3tc2*-null mice developed an early onset progressive peripheral neuropathy with decreased motor and sensory nerve conduction velocity and hypomyelination. Onset of myelin pathology in sciatic nerves of *Sh3tc2*^−/−^ mice was somewhere between postnatal Days 0 and 5, since at 5 days of age there was already a difference between the mutants and controls (Arnaud *et al.*, 2009; Gouttenoire *et al.*, 2013). This phenotype was progressive as demonstrated by increasing myelin pathology and more difference in nerve conduction velocities in mutant compared to control mice at 1 year of age as compared to 1–2 months of age (Arnaud *et al.*, 2009).

Murine SH3TC2 is specifically expressed in Schwann cells and localized to the plasma membrane and to the perinuclear endocytic recycling compartment, suggesting a possible function in myelination and/or in regions of axoglial interactions. Ultrastructural analysis of myelin in the peripheral nerve of mutant mice showed abnormal organization of the node of Ranvier, a phenotype that was confirmed in CMT4C patient nerve biopsies. These findings suggested a role for the *SH3TC2* gene product in myelination and in the integrity of the node of Ranvier (Arnaud *et al.*, 2009). SH3TC2 interacts with ERBB2 and plays a role in the regulation of ERBB2 intracellular trafficking from the plasma membrane upon NRG1 activation while dysregulated NRG1/ERBB signalling was found in SH3TC2-deficient Schwann cells that may account for the disturbed axonal size sensing and the hypomyelination present in CMT4C patients (Gouttenoire *et al.*, 2013). Additionally, SH3TC2 was found to be an effector of the small GTPase Rab11, a key regulator of recycling endosome functions. Neuropathy-causing missense mutations in SH3TC2 disrupted this interaction (Stendel *et al.*, 2010), providing a likely mechanism for the progressive nature of CMT4C neuropathy.

In summary, both the clinical phenotype and molecular basis of CMT4C suggest loss of function of SH3TC2, which results in early onset and slowly progressive hypomyelination in the peripheral nerves. *Sh3tc2*^−/−^ mice recapitulate all major aspects of the disease. We have therefore developed a gene replacement strategy for CMT4C using a viral vector delivered intrathecally for Schwann cell-specific expression of human SH3TC2 (hSH3TC2). Treatment of *Sh3tc2*^−/−^ mice at the age of 3 weeks resulted in significant improvement of several pathological features indicating that this approach could be further developed to treat CMT4C patients.

## Materials and methods

### Cloning of lentiviral vectors

**Figure 1:**
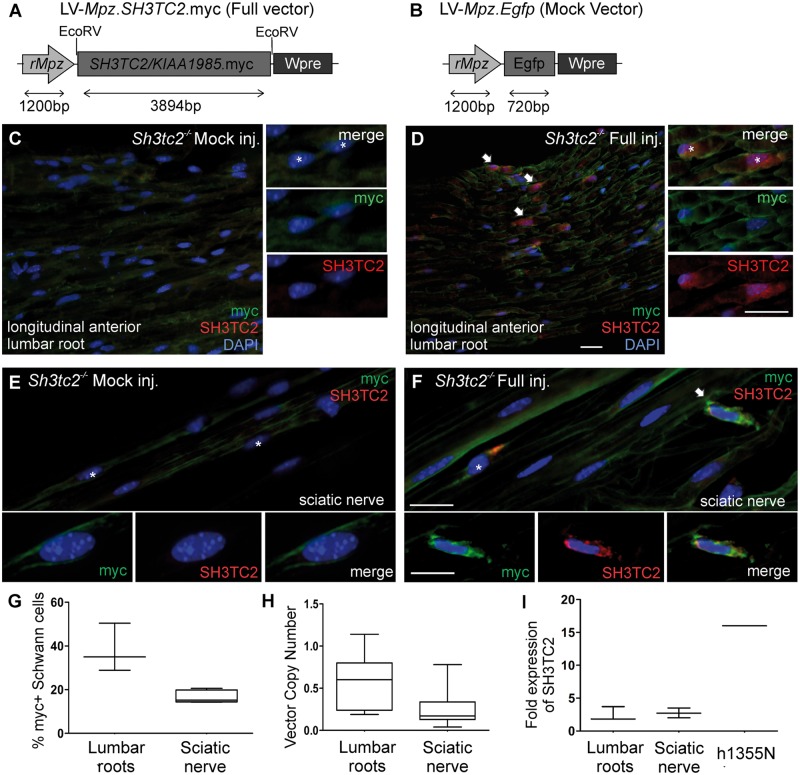
**Expression analysis of LV-*Mpz.SH3TC2.myc* vector.** Map of the LV-*Mpz.SH3TC2.myc* (**A**) and LV-*Mpz.Egfp* (**B**) transfer plasmids used for the full and mock lentiviral vector production, respectively. Longitudinal sections of lumbar roots (**C** and **D**) and sciatic nerves (**E** and **F**) from *Sh3tc2^−/−^* mice 4 weeks following intrathecal injection with the full or mock vector, as indicated, were stained with anti-myc (green) and anti-hSH3TC2 (red) antibodies. *Sh3tc2^−/−^* mice injected with full vector show hSH3TC2 expression in the perinuclear cytoplasm of a subset of Schwann cells co-localizing with myc immunoreactivity in lumbar roots (**D**) and sciatic nerve teased fibres (**F**). Human SH3TC2 expression was absent from mock vector-injected tissues (**C** and** E**). Higher magnification images and separate channels of expressing Schwann cells are shown. Cell nuclei were stained with DAPI (blue, asterisks). Scale bars = 20 μm. (**G**) Counts of myc-positive cells in sciatic nerves and lumbar roots of *Sh3tc2*^−/−^ mice 4 weeks following intrathecal injection of full vector were on average 38 ± 11.11% in lumbar roots (*n = *3) and 17 ± 2.88% in sciatic nerves (*n = *5 mice). (**H**) Vector copy numbers following intrathecal injection obtained from *n = *4 *Sh3tc2*^−/−^ mice 4 weeks after injection were on average 0.65 ± 0.21 in lumbar roots and 0.25 ± 0.15 in sciatic nerves. (**I**) Quantitative real-time PCR analysis of h*SH3TC2* expression in lumbar roots and sciatic nerves following intrathecal injection with full vector (*n = *3 mice)*. *Fold expression represents relative human *SH3TC2* mRNA expression levels of full vector-injected compared to non-injected *Sh3tc2*^−/−^ mice used as a baseline control. A human nerve sample h1355N (sural nerve biopsy) served as positive control. All samples were normalized to endogenous control *Gapdh*.

*SH3TC2* cDNA was cloned into the pcDNA3 vector under the CMV promoter (gift from Dr Vincenzo Lupo, University of Valencia). We added the myc-tag downstream of *SH3TC2* by PCR amplification (Supplementary material). Correct localization of SH3TC2.*myc* tag was confirmed by cell transfection into HeLa cells (Supplementary Fig. 1). The lentiviral vector backbone originating from pCCLsin.PPT.hPGK.GFP.pre, in which hPGK promoter was replaced by the myelin-specific rat myelin protein zero (*Mpz*) promoter (Sargiannidou *et al.*, 2015; Kagiava *et al.*, 2016) was used to clone the SH3TC2.*myc* construct. The Gibson Assembly master mix (NEB) was used to ligate the two fragments together. The full vector (LV-*Mpz.SH3TC2.myc*) was confirmed by direct sequencing analysis (Fig. 1A). The LV-*Mpz.EGFP* vector, already cloned in our lab, was used as a mock vector (Fig. 1B).

### Vector production

Lentiviral vectors were produced by transient co-transfection of HEK 293T cells with transfer plasmid and helper plasmids using the calcium phosphate co-precipitation method as described previously (Sargiannidou *et al.*, 2015) (Supplementary material).

### CMT4C mouse model

The generation and characterization of the *Sh3tc2*^−/−^ mouse model has been described previously (Arnaud *et al.*, 2009). This line was kept under specific pathogen free (SPF), standard controlled conditions of temperature (21–23°C), humidity, air exchange and light cycle (12/12 h light/dark) and provided with standardized mouse diet and drinking water *ad libitum.* All experimental procedures were conducted in accordance with animal care protocols approved by the Cyprus Government’s Chief Veterinary Officer (project license CY/EXP/PR.L3/2017) according to national law and European guidelines (EC Directive 86/609/EEC).

### Human sural nerve biopsy material

As a positive control for hSH3TC2 expression derived from the viral vector, we used anonymized material from a diagnostic sural nerve biopsy tissue following informed consent by the patient under the Protocol ‘Translational Research-Neurogenetics Department’ in accordance with the Declaration of Helsinki, and approved by the National Bioethics Committee of Cyprus (EEBK/EP/2013/28).

### Intraneural and intrathecal vector delivery

Vector delivery was performed under anaesthesia either by direct intraneural injection (10 μl) into surgically exposed mid-sciatic nerves of 2-month-old mice (Sargiannidou *et al.*, 2015), or intrathecally (30 μl) into L5–L6 intervertebral space of 3-week-old mice (Kagiava *et al.*, 2016, 2018; Kagiava and Kleopa, 2018) (Supplementary material).

### Vector copy number determination

The average vector copy number per cell in DNA extracted from lumbar root and sciatic nerves was calculated as the total vector copy number divided by the total cell number (Geraerts *et al.*, 2006; Christodoulou *et al.*, 2016) (Supplementary material).

### Immunofluorescence

Mice were anaesthetized with avertin according to institutionally approved protocols, and then transcardially perfused with phosphate-buffered saline (PBS) followed by fresh 4% paraformaldehyde. The lumbar spinal cord with all roots attached as well as sciatic nerves were dissected and frozen for cryosections. Sciatic nerves were also teased into fibres under a stereoscope. Teased fibres or sections were permeabilized in cold acetone and incubated at room temperature with a blocking solution of 5% bovine serum albumin (BSA) containing 0.5% Triton^™^ X for 1 h. Primary antibodies (Supplementary material) were incubated at 4°C overnight followed by appropriate secondary antibodies at room temperature. Slides were mounted with fluorescent mounting medium (DAKO) and images photographed under a fluorescence microscope with a digital camera using AxioVision software (Carl Zeiss MicroImaging).

Quantification of SH3TC2/myc expression was performed at 4 weeks post-injection in ventral root sections (*n = *3 mice) and in sciatic nerve teased fibres (*n = *5 mice) from five different areas from each slide. The number of myc-positive Schwann cells as well as the total number of cells in each picture was counted to determine the average expression ratio.

### Immunoblot analysis

Immunoblots of lumbar root and sciatic nerve lysates collected 4 weeks post-injection (Supplementary material) were incubated with anti-myc (1:1000; Santa Cruz) and anti-GAPDH (1:4000; Santa Cruz Biotechnology) primary antibodies followed by HRP-conjugated anti-mouse or anti-rabbit secondary antibodies (Jackson ImmunoResearch, diluted 1:3000). The bound antibody was visualized by an enhanced chemiluminescence system (GE Healthcare Life Sciences).

### Real-time PCR analysis of SH3TC2 expression

RNA was isolated with the Qiagen RNeasy® Lipid Tissue Mini Kit following the manufacturer’s protocol from snap-frozen lumbar roots and sciatic nerve tissues 4 weeks after intrathecal injection as well as from a frozen human sciatic nerve biopsy sample used as positive control and from non-injected *Sh3tc2*^−/−^ mouse nerves used a baseline control. After DNase treatment, RNA was quantified by spectrophotometry and 0.5 μg of RNA was used to synthesize cDNA using TaqMan^TM^ reverse-transcription reagents. Human SH3TC2 probe (Hs00226194_m1, Applied Biosystems) was used.

### Treatment trial in *Sh3tc2*^−/−^ mice

For the gene therapy trial, we used two groups of *Sh3tc2^−/−^* mice. All animals were intrathecally injected at the age of 3 weeks. Littermate mice were randomized to either receiving the full vector LV-Mpz.*SH3TC2.myc* (treatment group) or the mock vector LV-Mpz.*Egfp* (lacking the *Sh3tc2 *gene) as control group. Randomization was based on animal numbering after tailing. We randomized 2:1 because more mice with full vector injection were needed to analyse SH3TC2.myc expression by immunostaining, immunoblot, and real-time PCR (above), therefore more fully-treated mice were available for behavioural and electrophysiological studies. However, an equal *n* number was used for the morphometric analysis groups (semithin sections). Mice were evaluated at the age of 11 weeks by behavioural analysis (*n = *12 mock and *n = *21 full vector) and electrophysiological testing (*n = *10 mock and *n = *17 full vector), and sacrificed afterwards for SH3TC2 expression analysis or for quantitative morphometric analysis (*n = *9–10 per group) (Supplementary Fig. 8). All outcome evaluations were performed by examiners blinded to the treatment condition. Primary endpoint was considered the rescue of pathological changes in sciatic nerves. Secondary endpoints were the improvement in motor conduction velocities and in behavioural performance.

### Behavioural analysis

Rotarod and foot grip tests were performed 8 weeks post-injection to assess motor balance, coordination and muscle strength as described previously (Kagiava *et al.*, 2018) (Supplementary material).

### Electrophysiological assessment

Bilateral sciatic nerves were stimulated in anaesthetized animals at the sciatic notch and distally at the ankle via bipolar electrodes with supramaximal square-wave pulses (5 V) of 0.05 ms. Compound muscle action potentials were recorded by a bipolar electrode inserted between digits 2 and 3 of the hind paw and latencies were measured from the stimulus artefact to the onset of the negative M-wave deflection based on previously described methodology (Zielasek *et al.*, 1996). MNCVs were calculated by dividing the distance between the stimulating and recording electrodes by the result of subtracting the distal latency from the proximal latency.

### Morphometric analysis of myelination in lumbar spinal roots and mid-sciatic nerves

Mice were transcardially perfused with 2.5% glutaraldehyde in 0.1 M phosphate buffer. The lumbar roots and sciatic nerves, were dissected and fixed overnight at 4°C, then osmicated, dehydrated, and embedded in araldite resin. Transverse semithin sections (1 μm) of the lumbar spinal cord with ventral roots and mid-sciatic nerves were obtained and stained with alkaline toluidine blue. Sections were visualized with 10×, 20×, 40×, and 63× objective lenses and captured with a Zeiss AxioCam HR camera. To examine the degree of abnormal myelination in both groups, images visualized at 63× were used.

The g-ratios for all myelinated fibres in the two treatment groups were calculated by Image-Pro software using a custom-made macro that detects the axons and their myelin sheath according to colour. This macro calculates g-ratio by dividing the average inner perimeter of the axon by the average outer perimeter of the axon, as well as the average myelin thickness for each myelinated fibre. This analysis allowed the calculation of g-ratios and myelin thickness. Because more pronounced myelination deficits were observed in fibres >4 μm in diameter in the original characterization of this model (Arnaud *et al.*, 2009), we also analysed the subset of fibres >4 μm separately in addition to the analysis of all fibres >1 μm. In addition, completely demyelinated fibres (defined as axons >1 μm in diameter devoid of myelin sheath) in anterior lumbar roots and mid-sciatic nerves were manually counted and compared to the total fibre numbers, to obtain the ratio of demyelinated fibres.

### Assessment of nodal pathology

Teased sciatic nerves from wild-type (*n = *2), full vector-injected (*n = *5) and mock vector-injected *Sh3tc2^−/−^* mice (*n = *4) were used to measure the nodal length by directly measuring the width of Na_v_1.6 immunoreactive area using Image-Pro software. A total of 55 nodes from mock-treated and 89 nodes from fully-treated mice, along with 18 nodes from wild-type mice were measured and compared.

### Assessment of transcriptional changes in treated *Sh3tc2*^−/−^ mice

The expression of several genes previously reported to be dysregulated in younger *Sh3tc2*^−/−^ mice (Arnaud *et al.*, 2009) was analysed in sciatic nerves and lumbar roots first in untreated 11-week old *Sh3tc2*^−/−^ compared to wild-type littermates (Supplementary material). Subsequently the expression of the most significantly dysregulated genes *Scip*, *Cdgap*, *Cxcl24* and *Mpz* was compared between full and mock treatment groups, using quantitative PCR. *Gapdh* was used as a loading control.

### Measurement of blood neurofilament light levels

Blood samples were obtained from 6- and 25-week-old wild-type (*n = *2–3 per age group) and untreated *Sh3tc2^−/−^* mice (*n = *3–4 per age group), as well as from 11-week-old full vector- or mock vector-treated *Sh3tc2*^−/−^ mice (*n = *5–7 per treatment group) using standard methods (Parasuraman *et al.*, 2010). Blood samples were collected into EDTA-containing tubes and centrifuged at 20°C and 3500 rpm for 10 min. Plasma was aliquoted and stored at −80°C until testing. Plasma sample neurofilament light (NfL) concentration was measured at UCL using a commercially available NF-Light kit on a single molecule array (Simoa^TM^) HD-1 instrument (Quanterix).

### Statistical analysis

Behavioural, electrophysiological and morphometric analysis data, as well as plasma NfL levels obtained from full or mock vector-treated groups were compared using the Mann-Whitney U-test. Nodal width, real time PCR results, and baseline NfL levels were compared with two-tailed Student’s *t*-test (significance level for all comparisons, *P* < 0.05).

### Data availability

Data supporting these findings are available upon request.

## Results

### The LV-*Mpz.SH3TC2.myc* vector induces widespread expression of SH3TC2 in Schwann cells

The LV-*Mpz.SH3TC2.myc* (full) and LV-*Mpz.Egfp* (mock) plasmids (Fig. 1A and B) were successfully cloned and verified by direct sequencing. Following lentiviral (LV) vector production, the vector titres obtained ranged from 5.3 × 10^9 ^to 1.5 × 10^11^ vector copies/ml as determined by quantitative PCR and ELISA. To assess expression and cell specificity based on the *Mpz* promoter, vectors were injected either intraneurally (10 μl) into exposed sciatic nerves (Sargiannidou *et al.*, 2015), or intrathecally (30 μl) into the L5–L6 intervertebral space of anaesthetized mice (Kagiava *et al.*, 2016, 2018). The expression and distribution of SH3TC2.myc was examined 4 and 8 weeks after vector injection of the full vector into *Sh3tc2*^−/−^ mice, compared to non-injected or mock vector-injected animals.

Immunostaining of sciatic nerve teased fibres from mice injected intraneurally with the LV-*Mpz.SH3TC2.myc *vector showed myc immunoreactivity in the perinuclear cytoplasm of a subset of myelinating Schwann cells in different nerve fascicles obtained from both proximal and distal sections of the nerve. In contrast, no myc immunoreactivity was detected in non-injected sciatic nerve fibres (Supplementary Fig. 2). We similarly examined the expression of myc-tagged SH3TC2 in sciatic nerve teased fibres from intrathecally-injected mice. We found perinuclear myc immunoreactivity in a subset of myelinating Schwann cells in spinal roots (Fig. 1C and D) and in peripheral nerves from full vector injected but not from mock vector injected mice, including sciatic nerve (Fig. 1E and F) and femoral motor nerve fibres (data not shown), in keeping with our previous findings showing widespread gene expression following intrathecal injection (Kagiava *et al.*, 2016). In addition to immunostaining with anti-myc antibodies, we tested several commercially available anti-SH3TC2 antibodies to confirm expression of the lentiviral vector and confirmed that a rabbit antiserum (ab204334) provided specific SH3TC2 immunoreactivity as demonstrated by immunostaining of human sural nerve biopsy sections (Supplementary Fig. 3).

Double immunostaining for hSH3TC2 and myc tag of lumbar root sections and sciatic nerve fibres from LV-*Mpz.SH3TC2.myc *intrathecally-injected *Sh3tc2*^−/−^ mice confirmed the co-localization of virally expressed hSH3TC2 in the perinuclear Schwann cell cytoplasm, typically in a granular pattern, in a subset of myelinating Schwann cells, while SH3TC2 immunoreactivity was absent in tissues from mock-injected *Sh3tc2*^−/−^ mice (Fig. 1C–F and Supplementary Fig. 4). To clarify the cell specificity of hSH3TC2 expression in peripheral nerve tissues further, we also stained sciatic nerve cross sections from LV-*Mpz.SH3TC2.myc *vector intrathecally-injected *Sh3tc2*^−/−^ mice with the combination of anti-SH3TC2 with anti-vimentin (fibroblast marker) or with myelin basic protein (MBP, a myelinating Schwann cell marker) antibodies. This analysis showed that virally delivered SH3TC2 was expressed specifically in MBP-positive myelinating Schwann cells but not in vimentin-positive fibroblasts or non-myelinating Schwann cells (Neuberger and Cornbrooks, 1989; Walko *et al.*, 2013) in the peripheral nerves of injected mice (Supplementary Fig. 5).

We used immunostaining to examine the expression of virally-delivered hSH3TC2 in relation to the recycling endosome GTPase Rab11, which is known to associate with SH3TC2 (Roberts *et al.*, 2010; Stendel *et al.*, 2010). Immunostaining for Rab11 in untreated *Sh3tc2*^−/−^ mice showed characteristic perinuclear expression, which did not differ from the Rab11 expression pattern in wild-type mouse nerve fibres. In LV-*Mpz.SH3TC2.myc*-injected *Sh3tc2*^−/−^ mice, virally expressed hSH3TC2 (labelled with anti-myc) showed co-localization with Rab11 in Schwann cells (Supplementary Fig. 6), indicating an interaction with endogenous Rab11, in keeping with the physiological role of SH3TC2.

Quantification of SH3TC2 expression rates revealed by immunostaining in *Sh3tc2*^−/−^ mice 4 weeks following intrathecal injection of LV-*Mpz-SH3TC2.myc* showed that 17 ± 2.9% of myelinating Schwann cells in sciatic nerves (*n = *5 mice counted) and 38.1 ± 11.1% in ventral lumbar roots (*n = *3 mice counted) expressed SH3TC2 (Fig. 1G). These expression rates corresponded to a proximal to distal gradient of vector biodistribution following intrathecal injection as assessed by higher vector copy numbers in lumbar roots (0.65 ± 0.21) compared to sciatic nerve (0.25 ± 0.15) samples (Fig. 1H).

Expression of myc-tagged hSH3TC2 was also shown by immunoblot analysis using the anti-myc antibody in sciatic nerve and lumbar root lysates obtained 4 weeks following intrathecal injection of the LV-*Mpz.SH3TC2.myc* vector. The predicted protein band at 144 kDa was detectable in sciatic nerves as well as in lumbar roots in two of the three mice examined compared to a negative control (Supplementary Fig. 7). Finally, hSH3TC2 expression was also assessed by quantitative PCR analysis. The *SH3TC2* transcript was detected in most sciatic nerve samples from either intraneurally (Supplementary Fig. 2E) or intrathecally-injected mice, as well as in lumbar root samples from intrathecally-injected animals (Fig. 1I), at levels that were below that of an adult human sural nerve.

### Gene replacement treatment in *Sh3tc2*^−/−^ mice leads to improved motor performance and nerve function

After establishing an efficient gene replacement method for SH3TC2, we proceeded with a randomized, mock vector-controlled, blinded treatment trial by intrathecal injection in groups of *Sh3tc2*^−/−^ littermate mice at 3 weeks of age, at early stages of neuropathy. Following injections, mice were observed for 8 weeks and then examined for possible phenotype rescue using behavioural, electrophysiological and histological methods at 11 weeks of age (Supplementary Fig. 8). Results were then compared between groups treated with the full (LV-*Mpz.SH3TC2.myc*) as opposed to the mock (LV-*Mpz.Egfp*) vectors. All behavioural and electrophysiological studies were performed by examiners blinded to the treatment status.

**Figure 2:**
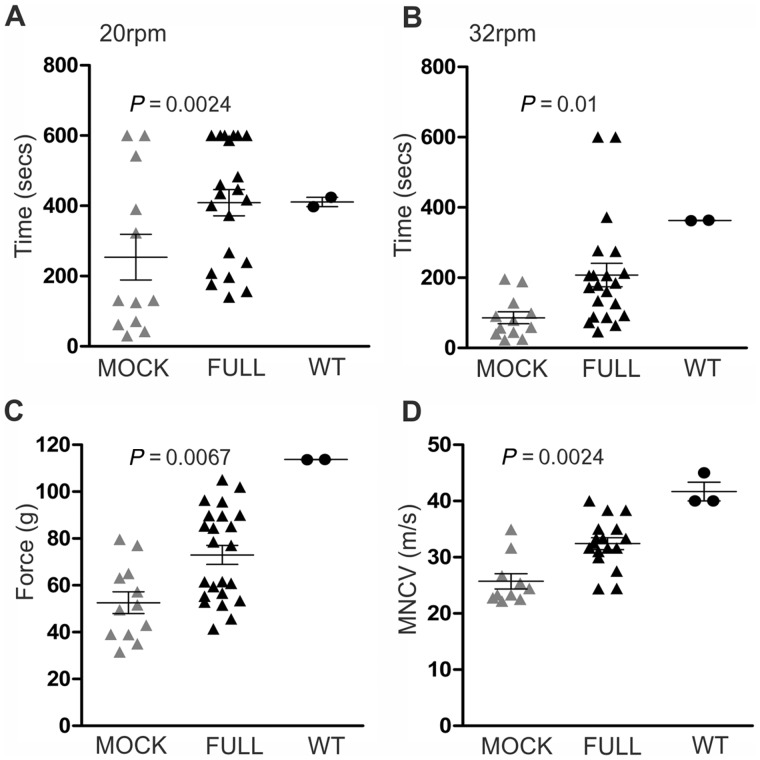
**Behavioural and electrophysiological analysis of treated *Sh3tc2^−/−^* mice.** Improved rotarod motor performance was observed at two different speeds tested, 20 rpm (**A**) and 32 rpm (**B**), in full vector-injected *Sh3tc2*^−/−^ mice (*n = *21) compared to mock vector-treated mice (*n = *12), as well as an increase in foot grip force generated in full (*n* = 23) compared to mock (*n* = 12) groups (**C**). Results of MNCV of mock and full vector-treated mice 8 weeks after vector injection are shown in **D**. Full vector-treated mice (*n = *17) show significantly increased MNCV compared to the mock group (*n = *10) (Mann-Whitney U-test used for all comparisons). Results from behavioural testing and MNCV measurements in wild-type (WT) littermate mice (*n = *2–3) are included in all diagrams, indicating that response to treatment in *Sh3tc2*^−/−^ mice was partial.

Rotarod analysis performed as described recently (Kagiava *et al.*, 2018) 8 weeks after injection showed that at a speed of 20 rpm, fully-treated mice (*n* = 21) remained on the rotarod significantly longer (408.8 ± 37.3 s) than mock-injected mice (253.9 ± 65 s; *P* = 0.024; *n* = 12). Likewise, at 32 rpm, fully-treated mice remained on the rotarod significantly longer than mock-treated littermates (full: 207.7 ± 33.4 s; mock: 91.3 ± 17.5 s; *P* = 0.01; Mann-Whitney U-test) (Fig. 2A and B). Furthermore, the foot grip test, which evaluates hind limb strength by using an equipment that automatically measures the grams of force required to pry the mouse from the handle, revealed that fully-treated mice (*n = *23) applied significantly greater force than mock-treated mice (*n = *12) (full: 91.4 ± 17 g; compared to mock: 62.2 ± 21 g; *P = *0.0067; Mann-Whitney U-test) (Fig. 2C). However, the motor performance of treated *Sh3tc2*^−/− ^remained below the indicative levels of wild-type littermate mice.

MNCV were measured in sciatic nerves according to previously published methods (Zielasek *et al.*, 1996). The mean MNCV in the fully-treated *Sh3tc2*^−/−^ mice (32.43 ± 4.36 m/s; *n* = 17 mice) was significantly faster than that in the mock-treated group (25.72 ± 4.29 m/s; *n* = 10 mice) (*P* = 0.0067; Mann-Whitney U-test), but remained slower than that of wild-type mice (41.7 ± 2.89 m/s; *n* = 3 mice) (Fig. 2D). Thus, both motor behavioural tests, as well as nerve conduction studies indicate a significant, albeit partial, therapeutic benefit in *Sh3tc2*^−/−^ mice treated with the LV-*Mpz.SH3TC2.myc* than mock vector-treated littermates.

### Improvement of peripheral myelination in treated *Sh3tc2*^−/−^ mice

To examine whether *SH3TC2* gene replacement can improve the hypo- and demyelination that characterizes *Sh3tc2*^−/−^ mice further, we performed morphological analysis of anterior lumbar roots and mid-sciatic nerves comparing treated (*n = *9) and mock-treated (*n = *10) *Sh3tc2*^−/−^ mice 8 weeks after vector injection, at 11 weeks of age. All morphometric analysis was performed blinded to the treatment status.

**Figure 3:**
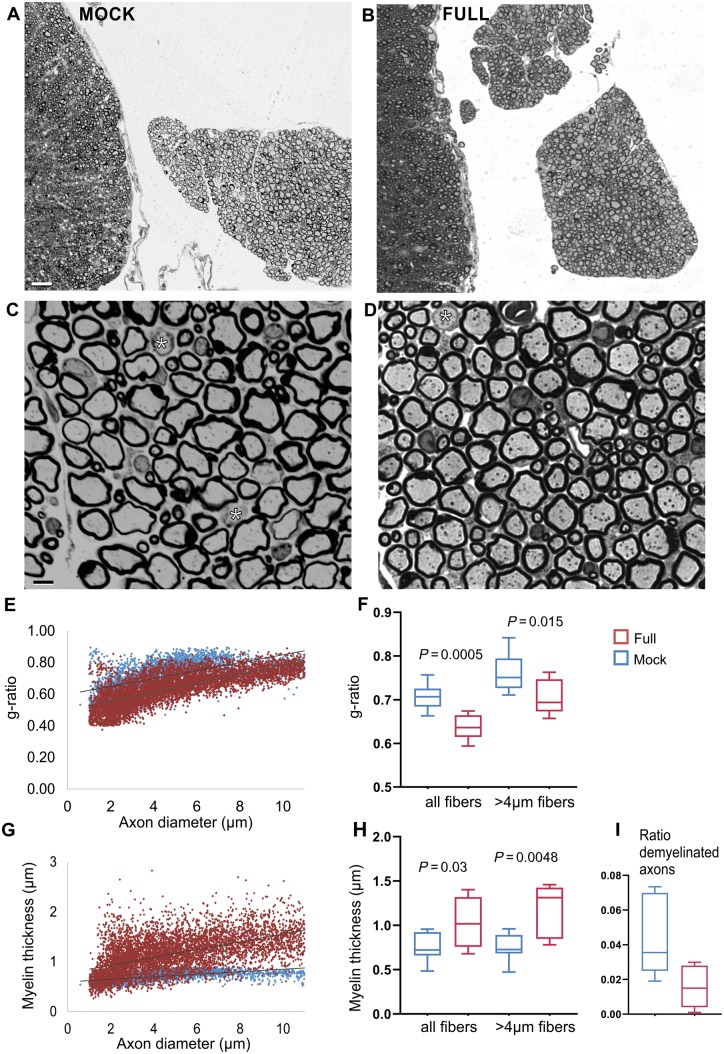
**Morphometric analysis of anterior lumbar roots. **Representative low (**A** and **B**) and higher magnification (**C** and **D**) images of toluidine blue-stained semithin sections from anterior lumbar spinal roots of 11-week-old mock- (**A** and **C**) and full vector- (**B** and** D**) treated *Sh3tc2^−/−^* mice. Mock-injected mice show thin myelin sheaths in most of the axons as well as some completely demyelinated fibres (asterisks in **C** and **D**), whereas full vector-injected mice show improvement of this myelin pathology. The scatter plots below display as indicated g-ratios (**E**) and myelin thickness (**G**) of individual axons versus axonal diameter (red points: full treatment group; light blue points: mock-treated group). Each point corresponds to one fibre. Comparison of g-ratios from all fibres, as well as from the subset of fibres >4 μm (**F**) shows significant reduction in fully-treated (*n = *9) compared to mock treated mice (*n = *10), while the corresponding myelin thickness (**H**) is increased. The ratio of completely demyelinated fibres is also reduced in fully-treated compared to mock-treated *Sh3tc2^−/−^* mice (**I**) (*P*-values obtained with the Mann-Whitney U*-*test). Scale bars = 20 μm (**A** and **B**), 2 μm (**C** and **D**).

For quantitative analysis of myelination, we measured the g-ratios as well as the average myelin thickness of all myelinated fibres with diameter >1 μm in toluidine blue stained transverse semithin sections (1 μm) using Image-Pro software. Because more pronounced myelination deficits were observed in fibres >4 μm in diameter in this model (Arnaud *et al.*, 2009), we also analysed the subset of fibres >4 μm separately in addition to the analysis of all fibres. Our morphological analysis confirmed a significant improvement in g-ratios, myelin thickness and ratios of demyelinated fibres in anterior lumbar roots and mid-sciatic nerves of fully-treated compared to mock-treated *Sh3tc2*^−/−^ mice. In ventral lumbar roots (Fig. 3), g-ratios of all myelinated fibres in mock-treated mice were 0.703 ± 0.028 as compared to 0.637 ± 0.027 in fully-treated mice (*P = *0.00052; Mann-Whitney U-test). In the subset of fibres >4 μm in diameter, the mock-treated group had a g-ratio of 0.762 ± 0.042 and the treated group 0.705 ± 0.038 (*P = *0.015). Average myelin thickness in mock-treated group was 0.766 ± 0.16 μm and in fully-treated 1.053 ± 0.28 μm (*P = *0.03), while in the subset of fibres >4 μm the myelin thickness was 0.758 ± 0.14 μm in the mock group and 1.185 ± 0.28 μm in the treated group (*P = *0.0048).

**Figure 4:**
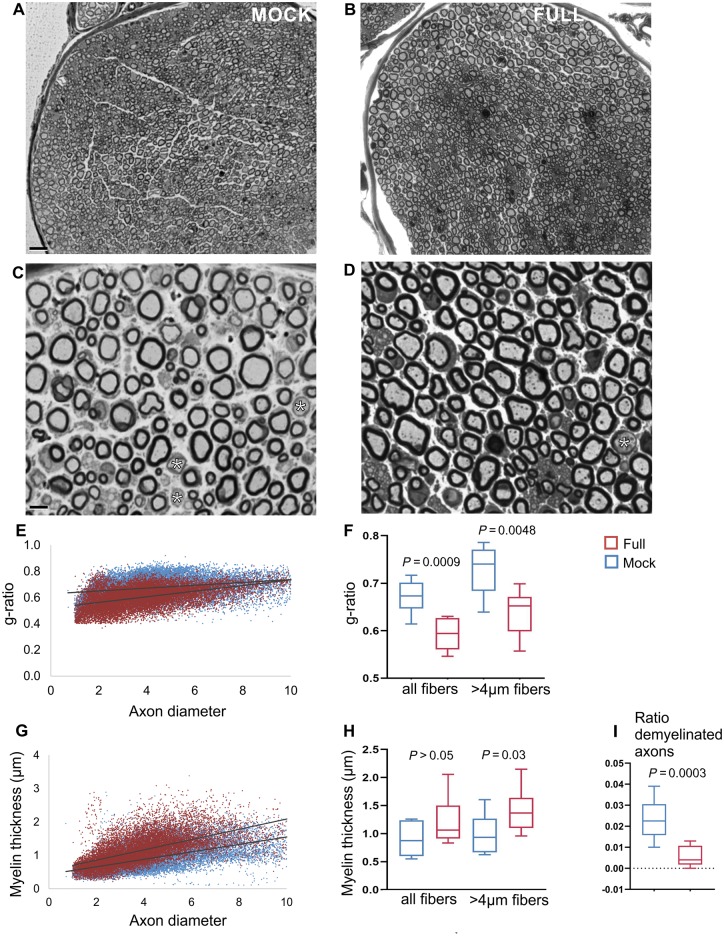
**Morphometric analysis of mid-sciatic nerves.** Representative low (**A** and **B**) and higher magnification (**C** and **D**) semithin sections from mid-sciatic nerves of 11-week-old mock- (**A** and **C**) and full vector- (**B** and** D**) treated *Sh3tc2^−/−^* mice. Mock-injected mice show thin myelin sheaths in most of the axons as well as some completely demyelinated fibres (asterisks in **C** and** D**), and these abnormalities are ameliorated in full vector injected mice. The scatter plots below display as indicated g-ratios (**E**) and myelin thickness (**G**) of individual axons versus axonal diameter (red points: full mice; light blue points: mock treated mice). Average g-ratios from all fibres, as well as from the subset of fibres >4 μm are shown in **F**, and corresponding average myelin thickness in **H**, demonstrating significant improvement of both parameters in full vector-treated (*n = *9) compared to mock-treated mice (*n = *10). There is also significant reduction of the ratio of completely demyelinated fibres in full vector-treated animals (**I**) (all *P*-values obtained with the Mann Whitney U*-*test). Scale bars = 20 μm (**A** and **B**), 2 μm (**C** and **D**).

In the sciatic nerves (Fig. 4), the average g-ratio of all myelinated fibres in the mock-treated group was 0.673 ± 0.034 and in the fully-treated group 0.593 ± 0.033 (*P = *0.0009), whereas for fibres >4 μm g-ratio was 0.725 ± 0.051 in the mock and 0.638 ± 0.046 in the treated group (*P = *0.0048). Myelin thickness of all size fibres was increased in treated mice (1.234 ± 0.407 μm) compared to the mock group (0.899 ± 0.311 μm), an increase that did not reach statistical significance (*P* > 0.05). However, in the larger diameter fibres (>4 μm) of fully-treated mice there was a significant increase in myelin thickness (1.410 ± 0.376 μm) compared to the mock group (0.986 ± 0.344 μm) (*P = *0.03).

Finally, the ratios of completely demyelinated fibres in both the anterior lumbar roots and in the sciatic nerves were significantly decreased in the fully-treated compared to the mock-treated mice (Figs 3I and 4I). Similar to the original observation (Arnaud *et al.*, 2009), we did not observe any axonal loss or any inflammatory cells in PNS tissues of *Sh3tc2*^−/−^ mice at 11 weeks of age, regardless of treatment condition. Moreover, we could not find any myelination defects in lumbar spinal cord fibres (data not shown) in keeping with lack of any CNS myelination defects in this model based on previous examination of the optic nerve (Arnaud *et al.*, 2009). Thus, LV-*Mpz.SH3TC2.myc*-treated mice show significant improvement of myelination in peripheral nervous system tissues compared to mock-treated littermates.

### Improvement of nodal pathological changes in treated *Sh3tc2^−/−^* mice

**Figure 5:**
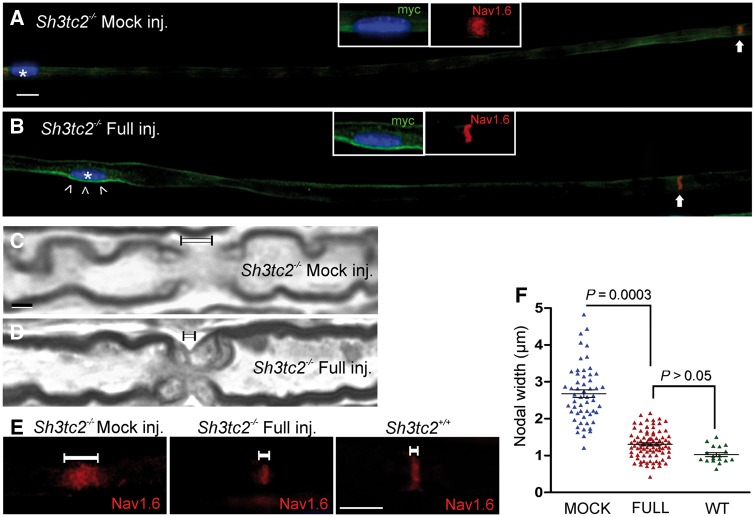
**Improvement of nodal phenotype in treated *Sh3tc2*^−/−^ mice. **Immunostaining of sciatic nerve teased fibres from mock (**A**) or full vector (**B**) treated *Sh3tc2*^−/−^ mice shows expression of myc-tagged SH3TC2 (green) only in treated fibres (open arrowheads in **B**) around the Schwann cell nucleus (asterisks) and the normal appearance of Na_v_1.6 (red)-labelled adjacent node in the fibre from fully-treated mouse (**B**) in contrast to the elongated Na_v_1.6-labelled node in the fibre from mock-treated mouse (**A**) (nodal areas indicated by arrows). (**C** and **D)** High magnification images of longitudinal sciatic nerve semithin sections from mock-injected (C) or full-vector injected (D) *Sh3tc2*^−/−^ mice demonstrate the nodal elongation (**C**), which improves after treatment (**D**). (**E**) Staining of sciatic nerve fibres from *Sh3tc2^+/+^* (wild-type, WT), as well as mock-treated and full vector-treated *Sh3tc2*^−/−^ mice with Na_v_1.6 (red) nodal marker shows the typical elongation of nodal width (indicated by white lines) in mock-treated *Sh3tc2*^−/−^ mice, which is improved in full vector-treated *Sh3tc2*^−/−^ mice nearing the appearance of *Sh3tc2^+/+^* nodal areas, consistent with improved nodal molecular architecture. (**F**) Results of quantification of 55 nodes from *n = *4 mock-treated and 89 nodes from *n = *5 full vector-treated *Sh3tc2*^−/−^ mice, along with 18 nodes from *n = *2 wild-type mice, confirm significant improvement of nodal elongation measured by Na_v_1.6 staining (Mann-Whitney U-test). Scale bars = 2 μm (**C** and **D**), 10 μm (**E**).

Since nodal abnormalities consisting of nodal widening have been detected as early as 1 month of age in *Sh3tc2^−/−^* mice and in biopsied nerves from CMT4C patients (Arnaud *et al.*, 2009), we also examined nodal pathology to clarify whether gene replacement could reverse some of these early onset pathological features of CMT4C. Morphometric analysis was performed in preparations of sciatic nerve teased fibres that were immunostained for nodal marker Na_v_1.6, as well as for paranodal Caspr (CNTNAP1) and juxtaparanodal K_v_1.1 (KCNA1) or Caspr2 (CNTNAP2). We found that the characteristic nodal widening present in nerve fibres from mock-treated animals was reduced and approached the wild-type appearance in nerve fibres of fully-treated *Sh3tc2^−/−^* mice with SH3TC2 expression (Fig. 5A–E and Supplementary Fig. 9). We assessed the nodal length by directly measuring the width of Na_v_1.6 immunoreactive area. Quantification of 55 nodes from *n = *4 mock-treated and 89 nodes from *n = *5 fully-treated *Sh3tc2^−/−^* mice, along with 18 nodes from *n = *2 wild-type mice confirmed these observations (Fig. 5F). Thus, this early characteristic nodal abnormality that occurs in the absence of SH3TC2 can also be rescued by the gene therapy.

### Transcriptional changes in *Sh3tc2^−/−^* mice

Several genes involved in myelination have been found to be downregulated in 4-week-old *Sh3tc2^−/−^* mice (Arnaud *et al.*, 2009) with simultaneous upregulation of transcription factors and other genes implicated in cell signalling and adhesion processes. These findings indicate that multiple genes and cellular pathways may play a role in CMT4C pathogenesis. Therefore, we examined whether *SH3TC2 *gene replacement could rescue some of these gene expression abnormalities. First, we examined the expression levels in lumbar roots as well as in sciatic nerves of selected dysregulated genes comparing 11-week-old *Sh3tc2^−/−^* mice with wild-type littermates. Several of these genes were upregulated in the sciatic nerves more than in the lumbar roots of *Sh3tc2^−/−^* mice compared to wild-type littermates, although mostly not at the same magnitude as reported at 4 weeks of age. Among the genes examined, the most prominent upregulation was found for Scip/Oct-6 (*Pou3f1*) expression, while we also found that *Mpz* expression was downregulated (Supplementary Fig. 10A). We then selected some of the most dysregulated genes at this age in *Sh3tc2^−/−^* mice, and performed a comparison of fully-treated versus mock-treated animals (*n = *4 mice per group). This analysis showed a trend for ameliorated overexpression of dysregulated genes in both lumbar roots and sciatic nerves from treated mice, including *Pou3f1*/Scip, *Cdgap*, and *Cxcl14*, but only *Pou3f1*/Scip downregulation in the sciatic nerves was statistically significant (Supplementary Fig. 10B and C).

### Blood neurofilament light levels as biomarkers

**Figure 6:**
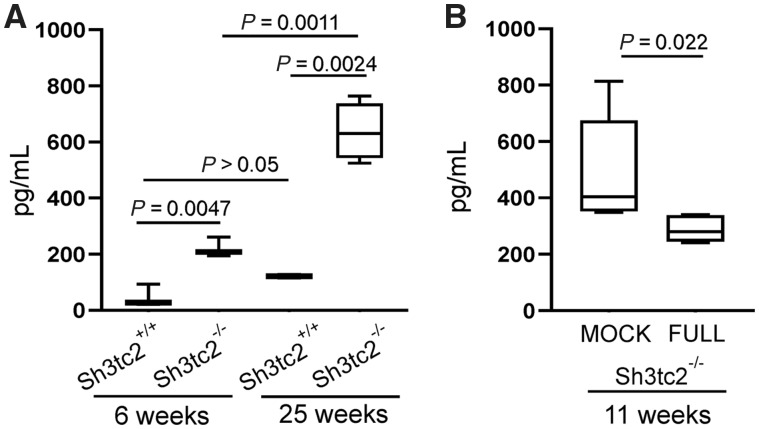
**Assessment of serum NfL levels in *Sh3tc2*^−/−^ mice at baseline and after treatment.** (**A**) Comparison of serum NfL levels between untreated *Sh3tc2*^−/−^ and *Sh3tc2*^+/+^ littermate mice showed significant elevation in the CMT4C model both at 6 weeks of age (*P = *0.0047; *n = *3 mice per group), as well as at 25 weeks of age (*P = *0.0024; *n = *4 *Sh3tc2*^−/− ^and *n = *2 *Sh3tc2*^+/+^ mice). The elevation of NfL concentration in *Sh3tc2*^−/−^ mice increased significantly from 6 to 25 weeks of age (*P = *0.0011), indicating progressive axonal dysfunction in this model of demyelinating neuropathy over time. In contrast, no significant progression was found when comparing 6- to 25-week old *Sh3tc2*^+/+^ mice (*P* > 0.05) (Student’s *t*-test for all baseline comparisons). (**B**) Comparison of NfL levels in 11-week old full vector-treated (FULL, *n = *7) compared to mock vector-treated (MOCK, *n = *5) *Sh3tc2*^−/−^ mice showed a significant reduction of NfL concentration (*P = *0.022, Mann-Whitney U-test).

Finally, in a small group of full or mock vector-treated *Sh3tc2^−/−^* mice (*n = *5–7 mice per group), we measured blood NfL levels, an established biomarker for axonal injury/degeneration found to be elevated in sera from CMT patients including two patients with CMT4C (Sandelius *et al.*, 2018). We first confirmed that serum NfL levels were elevated in *Sh3tc2^−/−^* compared to *Sh3tc2^+/+^* littermate mice starting at 6 weeks of age and showing progressive elevation at 25 weeks of age (Fig. 6A). In treatment groups, mock-treated *Sh3tc2^−/−^* mice at the age of 11 weeks showed an average NfL concentration of 491 ± 87 pg/ml (range: 348–814), while NfL concentration in full vector-treated *Sh3tc2^−/−^*mice was significantly lower 289 ± 18 pg/ml (range: 240–340; *P = *0.022; Mann-Whitney U-test) (Fig. 6B). Thus, this emerging blood biomarker shows a treatment response in the CMT4C model.

## Discussion

In this study, we demonstrate that a viral vector allowing for Schwann cell-targeted expression of hSH3TC2 could provide a gene therapy approach for CMT4C. Delivery of this vector into *Sh3tc2*^−/−^ mice, an authentic genetic model of CMT4C, achieved Schwann cell-specific expression that was widespread after intrathecal injection. An early treatment trial comparing LV-*Mpz.SH3TC2myc*-treated with mock vector-treated *Sh3tc2*^−/−^ mice demonstrated significant phenotype rescue, as indicated by improved motor behavioural tests, increased motor conduction velocities, reduction of myelin pathology and restoration of lengthened nodal architecture in *Sh3tc2*^−/−^ mice. Thus, our study provides a first proof of principle for viral gene replacement therapy targeted to Schwann cells to treat CMT4C.

Like other demyelinating neuropathies, CMT4C results from mutations in a Schwann cell-specific gene. Indeed the initial characterization of endogenous SH3TC2 expression in mice revealed high expression specifically in sciatic nerves, localized to Schwann cells (Arnaud *et al.*, 2009), indicating a cell-autonomous loss of function mechanism underlying CMT4C neuropathy. Therefore, a targeted replacement of SH3TC2 in myelinating Schwann cells will be ultimately necessary to treat CMT4C. Based on our previously-tested lentiviral vector with demonstrated Schwann cell tropism showing high infection rates in Schwann cells, as well as the rat *Mpz* promoter, which drives gene expression restricted to myelinating Schwann cells (Sargiannidou *et al.*, 2015; Kagiava *et al.*, 2016), we developed a novel vector to deliver the human *SH3TC2* gene into Schwann cells. Cell-specific expression of SH3TC2 was shown with immunostaining in combination with cell markers, as well as by immunoblot analysis and by detection of the transcript at the RNA level. Moreover, we could detect the characteristic vesicular pattern of SH3TC2 immunoreactivity at the plasma membrane and in perinuclear Schwann cell cytoplasm characteristic of the endocytic recycling compartment, as well as co-localization with the interacting protein Rab11 (Ullrich *et al.*, 1996; Arnaud *et al.*, 2009; Stendel *et al.*, 2010), suggesting that it assumes the functional role of the endogenous mouse SH3TC2.

The overall vector copy numbers and gene expression rates and levels achieved were not high, as only a subset of Schwann cells was found to be positive for SH3TC2/myc immunoreactivity. This is in line with the fact that we only achieved a partial phenotype rescue as demonstrated by all outcome measures. In other studies we also found that similar expression rates and lower than physiological expression levels of the neuropathy-associated gene such as *GJB1* (connexin 32) were still adequate to provide a significant therapeutic benefit in disease models, although not a complete rescue (Kagiava *et al.*, 2016, 2018). Moreover, lower correction rates *in vivo* were shown to provide therapeutic benefit in gene editing studies for muscular dystrophy (Long *et al.*, 2014), overall indicating that a therapeutic benefit can be achieved even with suboptimal gene replacement.

The *Sh3tc2*^−/−^ mouse provides a relevant model of CMT4C as it recapitulates all major phenotypic features of the disease. Although motor behavioural studies were not previously performed at baseline in this model, we used both rotarod analysis and foot grip tests, which are sensitive for peripheral nerve dysfunction as shown in our previous studies (Kagiava *et al.*, 2016; Kagiava *et al.*, 2018) and in other models of demyelinating neuropathy (Zhao *et al.*, 2018). Both of these tests were sensitive enough to demonstrate a significant improvement in the treated compared to the mock-treated group. In keeping with these motor behavioural improvements, we also found that motor nerve conduction velocities were increased in treated *Sh3tc2*^−/−^ mice. In the initial characterization of this model, at the age of 10–12 weeks, corresponding to the age of our treatment outcome evaluation, average MNCV were ∼10 m/s slower compared to the wild-type average (Arnaud *et al.*, 2009). In our groups, treated *Sh3tc2*^−/−^ mice were ∼9.3 m/s slower than the wild-type average, but 6.7 m/s faster and significantly improved than mock-treated littermates.

Detailed morphometric analysis of myelinated fibres in spinal motor roots and mid-sciatic nerves demonstrated an improvement in myelination assessed by g-ratio calculation as well as by direct average myelin thickness measurements. Hypomyelination was documented in *Sh3tc2*^−/−^ mice as early as postnatal Day 5, becoming progressively worse at later ages (Arnaud *et al.*, 2009). This indicates that the neuropathy in this model and in CMT4C results from a combination of developmental and progressive hypomyelination. This is in line with the putative function of SH3TC2 in myelin biogenesis and maintenance, based on the regulation of cargo transport, including myelin proteins, through the recycling endosome (Ang *et al.*, 2004; Trajkovic *et al.*, 2006; Winterstein *et al.*, 2008). We analysed mice at the age of 11 weeks at which a marked myelination defect was apparent and found a significant reduction of g-ratios along with increased myelin thickness in treated mice. The morphological improvements following gene therapy were only partial, likely reflecting the fact that only a subset of myelinating Schwann cells expressed SH3TC2. However, it is notable that even early onset pathological changes can be improved following treatment. Since we treated mice already at 3 weeks of age, it remains to be shown whether treatment at a later time point, could also provide a therapeutic benefit.

In this regard, the observed improvement in the abnormalities of nodal architecture is particularly interesting. The elongation of nodes of Ranvier observed in CMT4C nerve biopsies as well as in the *Sh3tc2*^−/−^ mouse present already at early developmental stages (Arnaud *et al.*, 2009) likely contributes to the functional impairment and progression of neuropathy. The cause of this abnormality is unclear. A dual mechanism drives the formation of the nodes of Ranvier and clustering of sodium channels during development, consisting of the nodal adhesion system of neurofascin186/NrCAM and gliomedin, as well as of the paranodal axon-glial junctions formed by Caspr/Contactin-NF155 that restrict the lateral diffusion of nodal sodium channels (Feinberg *et al.*, 2010; Amor *et al.*, 2014). Given that Na_v_1.6 channels remain clustered within the elongated nodes (Arnaud *et al.*, 2009) and our own observations), the elongation of the nodes in CMT4C is likely to result from defects in lateral growth of Schwann cells during development, making the second mechanism less efficient. Interestingly, these defects appear to be reversible, since we observed a significant remodelling of nodal architecture approaching the structure of wild-type nodes in treated compared to mock-treated mice. This may actually be a more responsive therapeutic outcome compared to the myelin thickness at the internode, accounting for the functional improvements observed in our study.

Dysregulation of several genes associated with various cellular functions, such as transcription factors regulating Schwann cell maturation, signalling, adhesion, immunity, extracellular matrix and myelination have been reported in 4-week-old *Sh3tc2^−/−^* mice (Arnaud *et al.*, 2009). We confirmed that several of these genes were upregulated in the sciatic nerves more that in the lumbar roots of *Sh3tc2^−/−^* mice compared to wild-type littermates at 11 weeks of age, although to a lesser degree that reported at 4 weeks, suggesting that transcriptional abnormalities are more pronounced during development and persist in adulthood. We found significant improvement only of Scip/Oct-6 (*Pou3f1*) dysregulation in sciatic nerves of treated mice, while other genes showed a non-significant trend for normalization. This result may be explained by the fact that we only achieved a partial expression and pathology rescue based on the other outcome measures used, as well as due to smaller degree of transcriptional changes at this age compared to 4 weeks of age, making a therapeutic response more difficult to demonstrate. Scip/Oct-6 plays a crucial role in the regulation of promyelinating Schwann cell maturation before axonal wrapping (Bermingham *et al.*, 1996) and appears to be a sensitive marker in hypomyelinating and demyelinating neuropathies, correlating with the overall reduction of promyelinating Schwann cells after SH3TC2 replacement. In a recent treatment trial in models of CMT1A, Scip/Oct-6 was also most prominently upregulated at baseline and was the most responsive to the therapeutic intervention (Zhao *et al.*, 2018).

Lastly, we demonstrate for the first time that a clinically relevant biomarker, plasma NfL concentration, previously shown to be elevated in CMT patients including two cases with CMT4C, correlating with clinical severity (Sandelius *et al.*, 2018), is also progressively elevated in *Sh3tc2^−/−^* mice and shows a significant improvement following gene replacement therapy. Thus, plasma NfL level appears to be a clinically relevant and treatment-responsive biomarker in the CMT4C model that can be useful in future preclinical and clinical studies.

Several challenges remain in the path to developing a clinically translatable gene therapy for CMT4C. In the current study, we tested the efficacy of intrathecal genes as early as possible, at 3 weeks of age. However, we also need to demonstrate that treatment at later stages after the onset of neuropathy can provide a similar therapeutic benefit. This question is relevant to treating CMT4C patients with a long disease course and likely ensuing secondary axonal loss, which is not present in the mouse model. Nevertheless, given that this is an early onset hypomyelinating neuropathy, even early intervention provides a proof of principle that abnormalities of myelin biogenesis and maintenance may be reversible, as shown by the rescue of the early nodal elongation and hypomyelination.

Furthermore, better vector biodistribution and higher gene expression levels are likely to provide a more robust therapeutic response. Alternative vectors such as AAV9 or AAVrh10 (Foust *et al.*, 2009; Tanguy *et al.*, 2015; Gurda *et al.*, 2016) could be tried if they can target Schwann cells as well as the lentiviral vector. They offer the advantage of improved safety with *in vivo* delivery because they do not integrate into the host genome and show low immunogenicity (Calcedo and Wilson, 2013). However, the limitation of smaller transgene capacity needs to be overcome, for example by using minimal Schwann cell-specific promoters. Confirmation of adequate viral vector biodistribution after intrathecal injection will also be needed in large animal models, while the alternative route of intravenous vector delivery should be considered (Bevan *et al.*, 2011; Tanguy *et al.*, 2015). This may be also relevant since the intrathecal injection could be difficult in CMT4C patients with prominent scoliosis (Kessali *et al.*, 1997; Gabreels-Festen *et al.*, 1999; Azzedine *et al.*, 2006).

In conclusion, this study provides evidence that *SH3TC2* gene replacement using a Schwann cell targeted viral vector can provide a therapeutic benefit in a model of CMT4C demyelinating neuropathy, leading to partial rescue of functional, electrophysiological and morphological abnormalities. Further studies are needed to optimize vector safety and biodistribution, gene expression levels, as well as to demonstrate effectiveness of this approach after the onset of neuropathy, in order to facilitate the clinical translation for CMT4C patients.

## Supplementary Material

Supplementary Data

## Abbreviations

CMT4: Charcot-Marie-Tooth disease type 4
MNCV: motor nerve conduction velocity
NfL: neurofilament light

## References

[awz064-B1] Amor V, Feinberg K, Eshed-Eisenbach Y, Vainshtein A, Frechter S, Grumet M et al Long-term maintenance of Na+ channels at nodes of Ranvier depends on glial contact mediated by gliomedin and NrCAM. J Neurosci 2014; 34: 5089–98. 24719088 10.1523/JNEUROSCI.4752-13.2014 PMC3983794

[awz064-B2] Ang AL, Taguchi T, Francis S, Folsch H, Murrells LJ, Pypaert M et al Recycling endosomes can serve as intermediates during transport from the Golgi to the plasma membrane of MDCK cells. J Cell Biol 2004; 167: 531–43. 15534004 10.1083/jcb.200408165 PMC2172492

[awz064-B3] Arnaud E, Zenker J, de Preux Charles AS, Stendel C, Roos A, Medard JJ et al SH3TC2/KIAA1985 protein is required for proper myelination and the integrity of the node of Ranvier in the peripheral nervous system. Proc Natl Acad Sci USA 2009; 106: 17528–33. 19805030 10.1073/pnas.0905523106 PMC2765159

[awz064-B4] Azzedine H, Ravise N, Verny C, Gabreels-Festen A, Lammens M, Grid D et al Spine deformities in Charcot-Marie-Tooth 4C caused by SH3TC2 gene mutations. Neurology 2006; 67: 602–6. 16924012 10.1212/01.wnl.0000230225.19797.93

[awz064-B5] Bermingham JR Jr, Scherer SS, O’Connell S, Arroyo E, Kalla K, Powell FR et al tst-1/SCIP/Oct-6 regulates a unique step in peripheral myelination and is required for normal respiration. Genes Dev 1996; 10: 1751–62. 8698235 10.1101/gad.10.14.1751

[awz064-B6] Bevan AK, Duque S, Foust KD, Morales PR, Braun L, Schmelzer L et al Systemic gene delivery in large species for targeting spinal cord, brain, and peripheral tissues for pediatric disorders. Mol Ther 2011; 19: 1971–80. 21811247 10.1038/mt.2011.157 PMC3222525

[awz064-B7] Calcedo R, Wilson JM Humoral immune response to AAV. Front Immunol 2013; 4: 341. 24151496 10.3389/fimmu.2013.00341 PMC3799231

[awz064-B8] Christodoulou I, Patsali P, Stephanou C, Antoniou M, Kleanthous M, Lederer CW Measurement of lentiviral vector titre and copy number by cross-species duplex quantitative PCR. Gene Ther 2016; 23: 113–8. 26202078 10.1038/gt.2015.60 PMC4705430

[awz064-B9] Colomer J, Gooding R, Angelicheva D, King RH, Guillen-Navarro E, Parman Y et al Clinical spectrum of CMT4C disease in patients homozygous for the p.Arg1109X mutation in SH3TC2. Neuromuscul Disord 2006; 16: 449–53. 16806930 10.1016/j.nmd.2006.05.005

[awz064-B10] Feinberg K, Eshed-Eisenbach Y, Frechter S, Amor V, Salomon D, Sabanay H et al A glial signal consisting of gliomedin and NrCAM clusters axonal Na+ channels during the formation of nodes of Ranvier. Neuron 2010; 65: 490–502. 20188654 10.1016/j.neuron.2010.02.004 PMC2831809

[awz064-B11] Foust KD, Nurre E, Montgomery CL, Hernandez A, Chan CM, Kaspar BK Intravascular AAV9 preferentially targets neonatal neurons and adult astrocytes. Nat Biotechnol 2009; 27: 59–65. 19098898 10.1038/nbt.1515 PMC2895694

[awz064-B12] Fridman V, Bundy B, Reilly MM, Pareyson D, Bacon C, Burns J et al CMT subtypes and disease burden in patients enrolled in the Inherited Neuropathies Consortium natural history study: a cross-sectional analysis. J Neurol Neurosurg Psychiatry 2015; 86: 873–8. 25430934 10.1136/jnnp-2014-308826 PMC4516002

[awz064-B13] Gabreels-Festen A, van Beersum S, Eshuis L, LeGuern E, Gabreels F, van Engelen B et al Study on the gene and phenotypic characterisation of autosomal recessive demyelinating motor and sensory neuropathy (Charcot-Marie-Tooth disease) with a gene locus on chromosome 5q23-q33. J Neurol Neurosurg Psychiatry 1999; 66: 569–74. 10209165 10.1136/jnnp.66.5.569 PMC1736348

[awz064-B14] Geraerts M, Willems S, Baekelandt V, Debyser Z, Gijsbers R Comparison of lentiviral vector titration methods. BMC Biotechnol 2006; 6: 34. 16836756 10.1186/1472-6750-6-34 PMC1534021

[awz064-B15] Gooding R, Colomer J, King R, Angelicheva D, Marns L, Parman Y et al A novel Gypsy founder mutation, p.Arg1109X in the CMT4C gene, causes variable peripheral neuropathy phenotypes. J Med Genet 2005; 42: e69. 16326826 10.1136/jmg.2005.034132 PMC1735969

[awz064-B16] Gouttenoire EA, Lupo V, Calpena E, Bartesaghi L, Schupfer F, Medard JJ et al Sh3tc2 deficiency affects neuregulin-1/ErbB signaling. Glia 2013; 61: 1041–51. 23553667 10.1002/glia.22493

[awz064-B17] Gurda BL, De Guilhem De Lataillade A, Bell P, Zhu Y, Yu H, Wang P et al Evaluation of AAV-mediated gene therapy for central nervous system disease in canine mucopolysaccharidosis VII. Mol Ther 2016; 24: 206–16. 26447927 10.1038/mt.2015.189 PMC4817811

[awz064-B18] Kagiava A, Karaiskos C, Richter J, Tryfonos C, Lapathitis G, Sargiannidou I et al Intrathecal gene therapy in mouse models expressing CMT1X mutations. Hum Mol Genet 2018; 27: 1460–73. 29462293 10.1093/hmg/ddy056

[awz064-B19] Kagiava A, Kleopa KA Intrathecal delivery of viral vectors for gene therapy. Methods Mol Biol 2018; 1791: 277–85. 30006718 10.1007/978-1-4939-7862-5_22

[awz064-B20] Kagiava A, Sargiannidou I, Theophilidis G, Karaiskos C, Richter J, Bashiardes S et al Intrathecal gene therapy rescues a model of demyelinating peripheral neuropathy. Proc Natl Acad Sci USA 2016; 113: E2421–9. 27035961 10.1073/pnas.1522202113 PMC4855595

[awz064-B21] Kessali M, Zemmouri R, Guilbot A, Maisonobe T, Brice A, LeGuern E et al A clinical, electrophysiologic, neuropathologic, and genetic study of two large Algerian families with an autosomal recessive demyelinating form of Charcot-Marie-Tooth disease. Neurology 1997; 48: 867–73. 9109869 10.1212/wnl.48.4.867

[awz064-B22] Lassuthova P, Mazanec R, Vondracek P, Siskova D, Haberlova J, Sabova J et al High frequency of SH3TC2 mutations in Czech HMSN I patients. Clin Genet 2011; 80: 334–45. 21291453 10.1111/j.1399-0004.2011.01640.x

[awz064-B23] LeGuern E, Guilbot A, Kessali M, Ravise N, Tassin J, Maisonobe T et al Homozygosity mapping of an autosomal recessive form of demyelinating Charcot-Marie-Tooth disease to chromosome 5q23-q33. Hum Mol Genet 1996; 5: 1685–8. 8894708 10.1093/hmg/5.10.1685

[awz064-B24] Long C, McAnally JR, Shelton JM, Mireault AA, Bassel-Duby R, Olson EN Prevention of muscular dystrophy in mice by CRISPR/Cas9-mediated editing of germline DNA. Science 2014; 345: 1184–8. 25123483 10.1126/science.1254445 PMC4398027

[awz064-B25] Lupo V, Galindo MI, Martinez-Rubio D, Sevilla T, Vilchez JJ, Palau F et al Missense mutations in the SH3TC2 protein causing Charcot-Marie-Tooth disease type 4C affect its localization in the plasma membrane and endocytic pathway. Hum Mol Genet 2009; 18: 4603–14. 19744956 10.1093/hmg/ddp427

[awz064-B26] Neuberger TJ, Cornbrooks CJ Transient modulation of Schwann cell antigens after peripheral nerve transection of subsequent regeneration. J Neurocytol 1989; 18: 695–710. 2515258 10.1007/BF01187088

[awz064-B27] Parasuraman S, Raveendran R, Kesavan R Blood sample collection in small laboratory animals. J Pharmacol Pharmacother 2010; 1: 87–93. 21350616 10.4103/0976-500X.72350 PMC3043327

[awz064-B28] Perez-Garrigues H, Sivera R, Vilchez JJ, Espinos C, Palau F, Sevilla T Vestibular impairment in Charcot-Marie-Tooth disease type 4C. J Neurol Neurosurg Psychiatry 2014; 85: 824–7. 24614092 10.1136/jnnp-2013-307421

[awz064-B29] Roberts RC, Peden AA, Buss F, Bright NA, Latouche M, Reilly MM et al Mistargeting of SH3TC2 away from the recycling endosome causes Charcot-Marie-Tooth disease type 4C. Hum Mol Genet 2010; 19: 1009–18. 20028792 10.1093/hmg/ddp565 PMC2830826

[awz064-B30] Sandelius A, Zetterberg H, Blennow K, Adiutori R, Malaspina A, Laura M et al Plasma neurofilament light chain concentration in the inherited peripheral neuropathies. Neurology 2018; 90: e518–e24. 29321234 10.1212/WNL.0000000000004932 PMC5818017

[awz064-B31] Sargiannidou I, Kagiava A, Bashiardes S, Richter J, Christodoulou C, Scherer SS et al Intraneural GJB1 gene delivery improves nerve pathology in a model of CMT1X. Ann Neurol 2015; 78: 303–16. 26010264 10.1002/ana.24441

[awz064-B32] Senderek J, Bergmann C, Stendel C, Kirfel J, Verpoorten N, De Jonghe P et al Mutations in a gene encoding a novel SH3/TPR domain protein cause autosomal recessive Charcot-Marie-Tooth Type 4C neuropathy. Am J Hum Genet 2003; 73: 1106–19. 14574644 10.1086/379525 PMC1180490

[awz064-B33] Stendel C, Roos A, Kleine H, Arnaud E, Ozcelik M, Sidiropoulos PN et al SH3TC2, a protein mutant in Charcot-Marie-Tooth neuropathy, links peripheral nerve myelination to endosomal recycling. Brain 2010; 133 (Pt 8): 2462–74. 20826437 10.1093/brain/awq168

[awz064-B34] Tanguy Y, Biferi MG, Besse A, Astord S, Cohen-Tannoudji M, Marais T et al Systemic AAVrh10 provides higher transgene expression than AAV9 in the brain and the spinal cord of neonatal mice. Front Mol Neurosci 2015; 8: 36. 26283910 10.3389/fnmol.2015.00036 PMC4516891

[awz064-B35] Trajkovic K, Dhaunchak AS, Goncalves JT, Wenzel D, Schneider A, Bunt G et al Neuron to glia signaling triggers myelin membrane exocytosis from endosomal storage sites. J Cell Biol 2006; 172: 937–48. 16520383 10.1083/jcb.200509022 PMC2063736

[awz064-B36] Ullrich O, Reinsch S, Urbe S, Zerial M, Parton RG Rab11 regulates recycling through the pericentriolar recycling endosome. J Cell Biol 1996; 135: 913–24. 8922376 10.1083/jcb.135.4.913 PMC2133374

[awz064-B37] Varley TL, Bourque PR, Baker SK Phenotypic variability of CMT4C in a French-Canadian kindred. Muscle Nerve 2015; 52: 444–9. 25737037 10.1002/mus.24640

[awz064-B38] Walko G, Wogenstein KL, Winter L, Fischer I, Feltri ML, Wiche G Stabilization of the dystroglycan complex in Cajal bands of myelinating Schwann cells through plectin-mediated anchorage to vimentin filaments. Glia 2013; 61: 1274–87. 23836526 10.1002/glia.22514

[awz064-B39] Winterstein C, Trotter J, Kramer-Albers EM Distinct endocytic recycling of myelin proteins promotes oligodendroglial membrane remodeling. J Cell Sci 2008; 121 (Pt 6): 834–42. 18303048 10.1242/jcs.022731

[awz064-B40] Zhao HT, Damle S, Ikeda-Lee K, Kuntz S, Li J, Mohan A et al PMP22 antisense oligonucleotides reverse Charcot-Marie-Tooth disease type 1A features in rodent models. J Clin Invest 2018; 128: 359–68. 29202483 10.1172/JCI96499 PMC5749515

[awz064-B41] Zielasek J, Martini R, Toyka KV Functional abnormalities in P_0_-deficient mice resemble human hereditary neuropathies linked to P_0_ gene mutations. Muscle Nerve 1996; 19: 946–52. 8756159 10.1002/(SICI)1097-4598(199608)19:8<946::AID-MUS2>3.0.CO;2-8

