# Supplementary material for: Gene replacement therapy in a model of Charcot-Marie-Tooth 4C neuropathy
Source: Brain. 2019 Mar 25;142(5):1227–41. doi: 10.1093/brain/awz064 (PMC6487329; doi:10.1093/brain/awz064)
Supplement: Supplementary Data [file awz064_supplementary_data.pdf]

SUPPLEMENTARY METHODS

Cloning of lentiviral vectors

SH3TC2 cDNA cloned in pcDNA3 under CMV promoter was used to add the myc-tag downstream of SH3TC2 cDNA by PCR amplification using forward and reverse primers as follows: *pcBspE-F* (5'- ctgagctccggattttcaataagctgacagagctgc-3'); *pcmyc-R2* (5'-tggccctcgagcagaagctgatcagcgaggaggacctgtaagatatccat-3'). A PCR product of 536bp was gel-extracted, purified (Gel extraction kit, Qiagen), digested with BspEI and EcoRV restriction enzymes, and the SH3TC2.*myc* tag was ligated into the pcDNA vector. Correct localisation of SH3TC2.*myc* tag was confirmed by cell transfection into Hela cells (**Supplementary Fig. 1**). The lentiviral vector backbone originating from pCCLsin.PPT.hPGK.GFP.pre, in which hPGK promoter was replaced by the myelin-specific rat myelin protein zero (*Mpz*) promoter was used to clone the SH3TC2.*myc* construct. The Gibson Assembly master mix (NEB) was used to ligate the two fragments together. The *SH3TC2.myc* sequence was PCR-amplified with *283G-F (5'-gacgctctgccaagcttgataccgatatccaagcttggtaccatcgcatggaatgggtggctgcttctgc-3')* and *283G-R (5'-gagcagaagctgatcagcgaggaggacctgtaagatatcaagcttatcgcgatacc-3').* Correct orientation and the in-frame positioning of the *Mpz* promoter and *SH3TC2.myc* in the full vector (LV-*Mpz-SH3TC2.myc*) was further confirmed by direct sequencing analysis (**Fig. 1A**).

Vector Production

A total of $5x10^6$ 293T cells were seeded in 10 cm plates 24 h prior to transfection in Iscove modified Dulbecco culture medium with 10% fetal bovine serum, penicillin (100 IU/ml), and streptomycin (100 mg/ml) in a 5% CO2 incubator. One hour prior to transfection the culture

medium was changed. A total of 64 μg of plasmid DNA was used for the transfection per dish: 16 μg of the envelope plasmid pMD2-VSVG, 16 μg of the packaging plasmid CMVΔR8.74 and 32 μg of the transfer vector plasmid. The precipitate was formed by adding the plasmids to a final volume of 540 μl and 60 μl of 2.5 M $CaCl_2$ and then adding drop wise 600 μl of 2x HEPES-buffered saline. The precipitate was added immediately to the cultures. The medium was then replaced after 6 h with fresh medium containing 1 mM Na butyrate. The conditioned medium was collected 60 h after transfection, cleared by low-speed centrifugation, and filtered through 0.22 μm-pore-size filters. The media was concentrated down from 6 ml to 250 μl volume by using the Lenti-X columns. Real time PCR and ELIZA were used to determine titer concentrations of the lentiviral vector which ranged from $5.3x10^9$ to $1.5x10^{11}$ before proceeding with gene delivery experiments.

Intraneural and intrathecal vector delivery

Intraneural vector delivery was performed by direct intraneural injection into surgically exposed mid-sciatic nerves distal to the sciatic notch, with pulled atraumatic glass pipettes filled with 10μl of the lentiviral vector using a microinjector, in order to minimize trauma to the nerves. For intrathecal injections, a small skin incision along the lower lumbar spine level was done to visualize the spine; the lentiviral vector was delivered into the L5-L6 intervertebral space of anesthetized mice. A flick of the tail was considered indicative of successful intrathecal administration. A 100-μL Hamilton syringe connected to a 30-gauge needle was used to inject 30 μL of lentiviral stock. Animals were euthanized 4 or 8 weeks post-injection, and dissected tissues were analysed by immunohistochemistry, immunoblotting, and RNA expression analysis.

Vector copy number determination

Genomic DNA was extracted from lumbar roots and sciatic nerves of mice 4 weeks after intrathecal vector delivery using the Qiagen DNA Mini (Thermo Fisher Scientific). The extracted DNA was analysed for yield and purity using a Nanodrop 1000 spectrophotometer. Approximately 20 ng of DNA were used as template for two quantitative PCR assays on an Applied Biosystems 7500 Real-Time PCR System involving 45 cycles of 15 s at 95 °C and 60 s at 60 °C. β-Actin-specific primers/probe targeting the mouse genome and WPRE-specific primer/probe targeting the WPRE gene, which is contained in the transgene, were used. Standard curves were created by serial dilution of quantified mouse genomic DNA, as well as quantified plasmid DNA containing the transgene cassette.

Immunofluorescence

Mice were anesthetized with avertin according to institutionally approved protocols, and then transcardially perfused with phosphate-buffered saline (PBS) followed by fresh 4% paraformaldehyde. The lumbar spinal cord with all roots attached as well as sciatic nerves were dissected and frozen for cryosections. Sciatic nerves were also teased into fibres under a stereoscope. Teased fibres or sections were permeabilised in cold acetone and incubated at room temperature (RT) with a blocking solution of 5% BSA containing 0.5% Triton-X for 1 h. Primary antibodies were incubated at 4°C overnight. Mouse monoclonal primary antibodies were used against myc (1:50; Santa Cruz), Caspr (1:100, Antibodies Incorporated) and vimentin (1:50; Millipore). Rabbit polyclonal antibodies were used against myelin basic protein (MBP; 1:300; gift from Prof. Richard Reynolds, Imperial College London), SH3TC2 (1:50, Abcam), Rab11 (Abcam, 1:50), Nav1.6 (1:100, Alomone), Kv1.1 (1:200, Alomone), Caspr2 (1:200, Alomone),

all diluted in blocking solution and incubated overnight at 4 °C. Slides were then incubated with fluorescein (FITC)-conjugated with goat anti-mouse and rhodamine (TRITC)-conjugated goat anti-rabbit secondary antibodies (1:500, Jackson ImmunoResearch) for 1h at RT. Cell nuclei were visualized with DAPI. Slides were then washed in PBS and incubated with fluorescein (FITC)-conjugated with goat anti-mouse and rhodamine (TRITC)-conjugated goat anti-rabbit secondary antibodies (1:500, Jackson ImmunoResearch) for 1h at RT. Cell nuclei were visualized with DAPI. Slides were mounted with fluorescent mounting medium (DAKO) and images photographed under a fluorescence microscope with a digital camera using Axiovision software (Carl Zeiss MicroImaging).

Immunoblot analysis

Fresh lumbar roots and sciatic nerves were collected at 4 weeks post-injection and lysed in ice-cold RIPA buffer (10 mM sodium phosphate, pH 7.0, 150 mM NaCl, 2 mM EDTA, 50 mM sodium fluoride, 1% Nonidet P-40, 1% sodium deoxycholate, and 0.1% SDS) containing a mixture of protease inhibitors (Roche). 150ng of lysed proteins were fractionated by 12% SDS/PAGE and then transferred to a Hybond-C Extra membrane (GE Healthcare Life Sciences) using a semidry transfer unit. Nonspecific sites on the membrane were blocked with 5% non-fat milk in PBS with Tween 20 (PBST) for 1 h at RT. Immunoblots were incubated with anti-myc (1:1,000; Santa Cruz) and anti-GAPDH (1: 4,000; Santa Cruz Biotechnology) antibodies at 4 °C overnight. After washing, the immunoblots were incubated with HRP-conjugated anti-mouse or anti-rabbit secondary antiserum (Jackson ImmunoResearch, diluted 1:3000) in 5% milk–PBST for 1 h. The bound antibody was visualized by an enhanced chemiluminescence system (GE Healthcare Life Sciences).

Behavioural analysis

Rotarod Test. Motor balance and coordination was determined using an accelerating rotarod apparatus (Ugo Basile). LV-*Mpz.SH3TC2.myc* and LV-*Mpz.Egfp*-treated mice were tested 8 weeks post-injection. Training of animals consisted of three trials per day with 15-min rest period between trials, for 3 consecutive days. Mice were placed on the rod, and the speed was gradually increased from 4 to 40 rotations per minute (rpm). The trial lasted until the mouse fell from the rod or after the mouse remained on the rod for 600 s and was then removed. Testing was performed on the fourth day using two different speeds, 20 and 32 rpm. The latency to fall was calculated for each speed.

Foot Grip Test. This test was performed 8 weeks post-injection. To measure grip strength, a mouse was held by the tail and lowered towards the apparatus (Ugo Basile, Italy) until it grabbed the grid first with all four paws and then with only the front paws. Mice were gently pulled back until they released the grid. Each session consisted of three consecutive trials. Measurements of the force in g were indicated on the equipment. Hindlimb force was calculated by subtracting the forelimb force from the force generated by all four limbs.

Results of all behavioral tests of LV-*Mpz.SH3TC2.myc* injected mice were compared to LV-*Mpz.Egfp* injected littermates using the Mann-Whitney *U* test. Significance was defined as $p < 0.05$ in all comparisons.

Assessment of transcriptional changes in treated *Sh3tc2*$^{-/-}$ mice

Bilateral sciatic nerves and lumbar spinal roots were dissected as above, fresh frozen and stored at -80˚C. RNA was extracted using the RNeasy Lipid Tissue Mini Kit according to the manufacturer’s instructions using the Qiazol Lysis Reagent followed by DNase treatment. RNA

samples were quantified by spectrophotometry (Nanodrop® ND—100) and subjected to reverse transcription (RT)-PCR (25˚C for 10 min, 48˚C for 30 min, and 95˚C for 5 min) using the TaqMan® RT-PCR Reagents and a GeneAmp® PCR System (Applied Biosystems, Singapore) (end volume of 40 μL containing 200 ng of RNA, dH2O, 10x Buffer, 25 mM MgCl2, dNTPs, random hexamers, RNase inhibitor, and reverse transcriptase). The expression of several genes previously reported to be dysregulated in *Sh3tc2*$^{-/-}$ mice was analysed first in untreated *Sh3tc2*$^{-/-}$ compared to 11-week old wild type mice (**Supplementary Fig. 10**). Subsequently, the expression of the most significantly dysregulated genes was compared between full and mock treatment groups using quantitative PCR. All probes used for quantitative PCR were obtained from TaqMan Gene Expression Assay. Control probe for GAPDH was included. Each sample was loaded in triplicate and contained 11.25ng of cDNA, 1 μL of TaqMan Gene Expression Assay, and 10 μL of TaqMan® Gene Expression Master Mix (end volume 20 μL). Samples were analysed using the 7900HT Fast Real-Time PCR System (hold at 55˚C for 2 min and at 95˚C for 10 min, followed by 40 cycles at 95˚C for 15 s and at 60˚C for 1 min). Data was normalized to GAPDH using the $2^{-\Delta\Delta CT}$ method.

SUPPLEMENTARY FIGURES AND LEGENDS

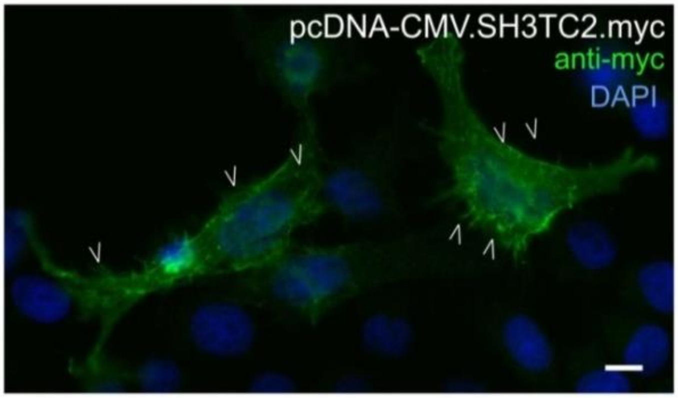

Supplementary Fig. 1: Expression of the myc-tagged SH3TC2 in Hela cells transfected with pcDNA-SH3TC2.myc and stained with anti-myc tag (green) and nuclear staining DAPI (blue). Myc-tagged SH3TC2 protein is localized around the nucleus and on the cell membrane in some of the cells (open arrows heads). Scale bar: 10µm

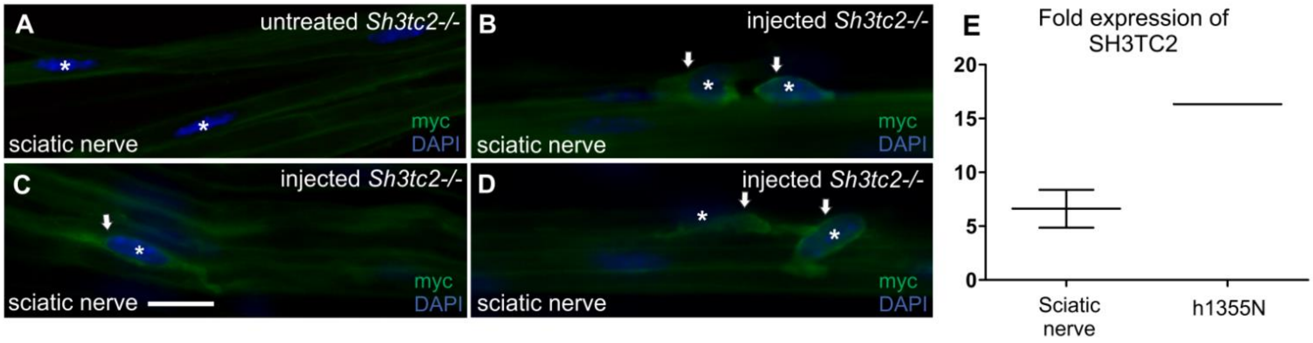

Supplementary Fig. 2: Expression of myc-tagged human SH3TC2 in sciatic nerve teased fibers of *Sh3tc2*$^{-/-}$ mice following *intraneural* injection of LV-*Mpz.SH3TC2*.myc. Teased fibers were stained with c-myc antibody (green) and counterstained with DAPI (asterisks, blue). Arrows point to myc-tagged hSH3TC2 expression in perinuclear Schwann cell cytoplasm in nerves of injected (**B-D**), but not of untreated *Sh3tc2*$^{-/-}$ mice (**A**). **E**: Real-Time PCR analysis of hSH3TC2 expression in sciatic nerves following intraneural injection of LV-*Mpz.SH3TC2.myc*. Fold expression represents relative hSH3TC2 mRNA expression levels of full vector-injected relative to non-injected *Sh3tc2*$^{-/-}$ mice. A human nerve sample h1355N (sural nerve biopsy) served as positive control. All samples were normalized to endogenous control GAPDH. Scale bar: 20μm

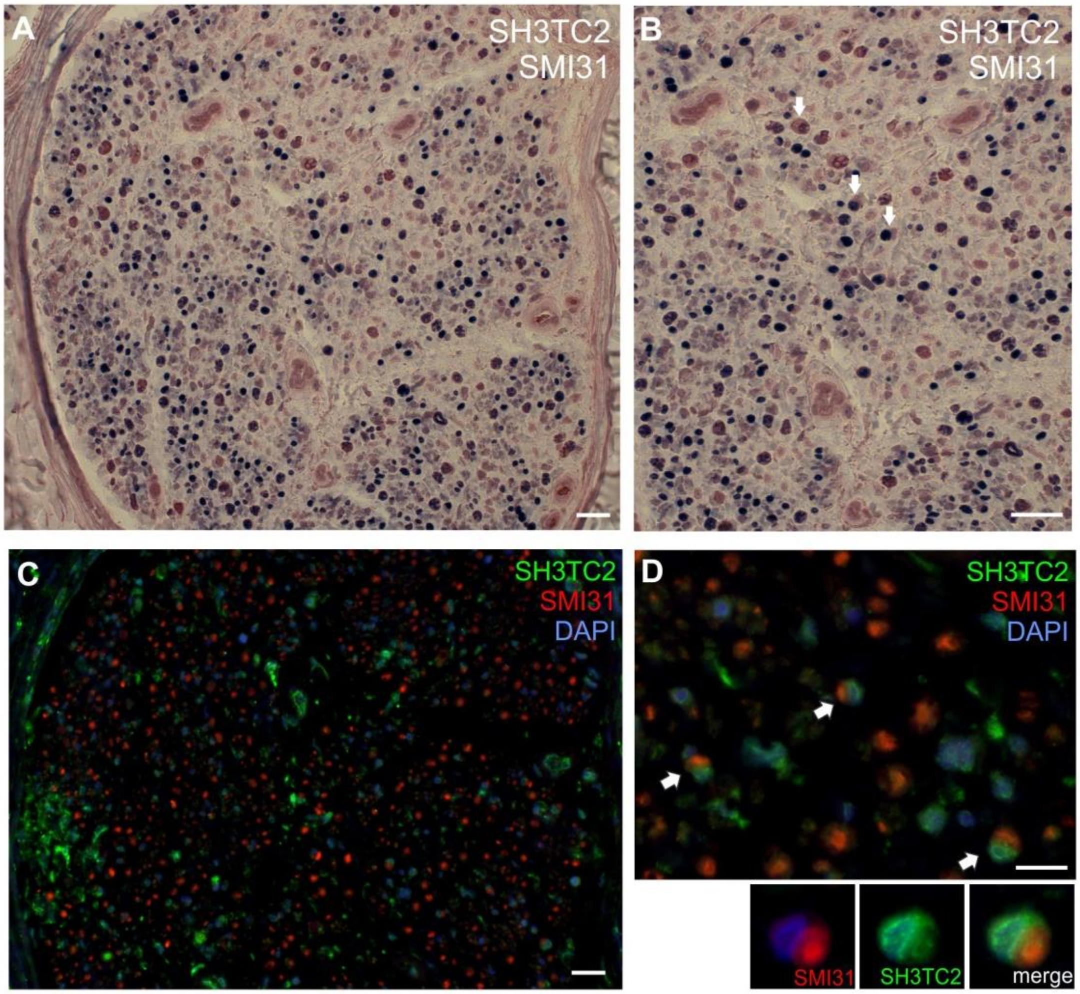

Supplementary Fig. 3: Validation of human-specific anti-SH3TC2 antibody on human sural nerve biopsy sections. Fixed sural nerve cross sections were double stained with anti-hSH3TC2 and neurofilament SMI31 (axonal marker). Immunohistochemistry (**A-B**) and immunofluorescence labelling (**C-D**) were used to localize the physiological SH3TC2 immunoreactivity in adult human nerve tissue. SH3TC2 (labelled brown in A-B and green in C-D) is expressed in the perinuclear Schwann cell cytoplasm (arrows in B, D) surrounding or adjacent to SMI31 labelled axons (stained blue in A-B and red in C-D). Cell nuclei are stained with DAPI (blue) in C-D. Details of individual myelinated fibers at the Schwann cell perinuclear area are shown at higher magnification in D. Scale bars in A and C: 30 μm, in B and D 10 μm.

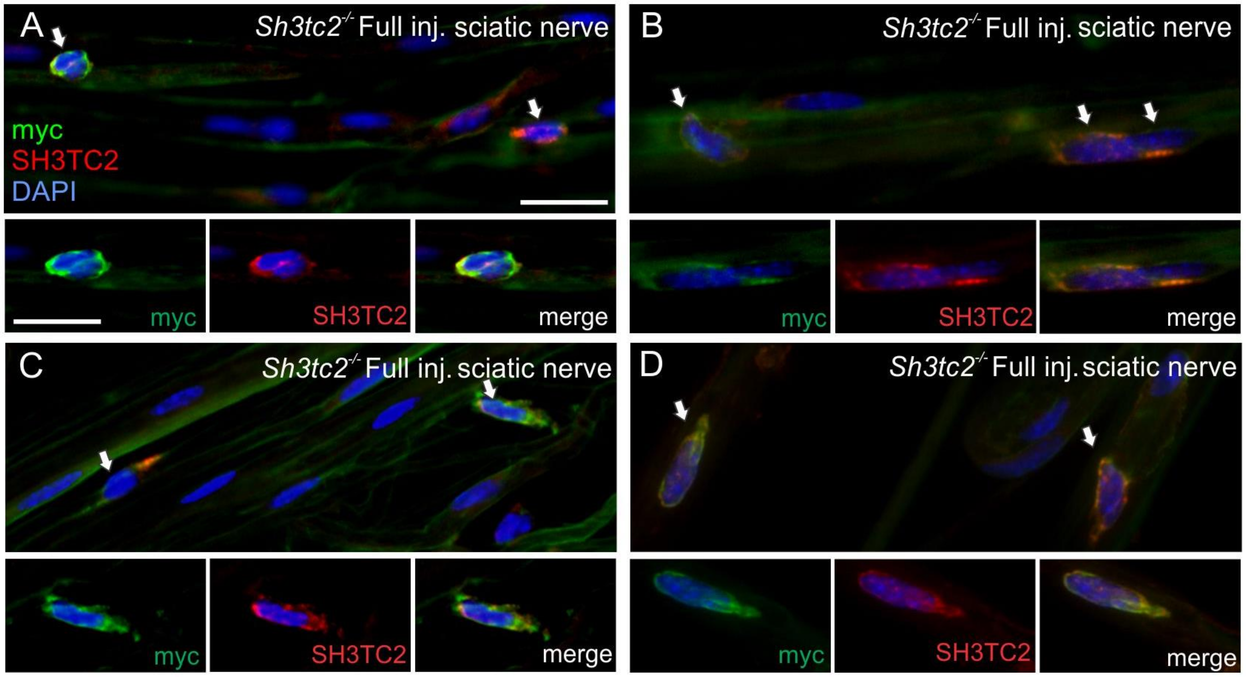

Supplementary Fig. 4: Expression of hSH3TC2 in sciatic nerve teased fibers. Sciatic nerve teased fibers from *Sh3tc2*$^{-/-}$ mice 4 weeks following intrathecal injection of LV-*Mpz.SH3TC2.myc* double immunostaining with the hSH3TC2 (red) and anti-myc (green) reveal the presence of myc-tagged hSH3TC2 protein (colocalisation of the two antibodies, **A-D** in the perinuclear Schwann cell cytoplasm in a granular-vesicular pattern (arrows). Higher magnification images of expressing Schwann cells are shown under the overview images. Scale bar: 20μm

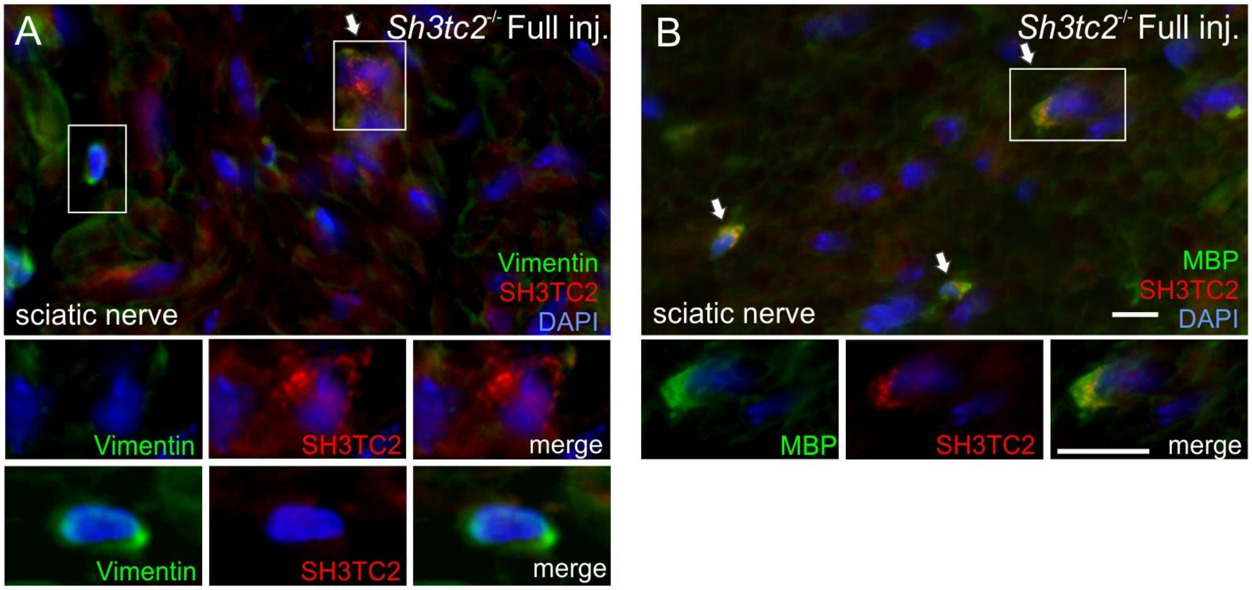

Supplementary Fig. 5: Expression of hSH3TC2 is specific to myelinating Schwann cells. Sciatic nerve cross sections from intrathecally injected *Sh3tc2*$^{-/-}$ mice immunostained with anti-SH3TC2 (red) and cell markers (green) vimentin (for fibroblasts) or myelin basic protein (MBP, for myelinating Schwann cells) shows that SH3TC2-expressing cells are vimentin-negative, while vimentin-positive fibroblasts do not express SH3TC2 (**A**). Furthermore, expression of SH3TC2 (arrows) is found in MBP-positive Schwann cells (**B**). Scale bars: 10 µm.

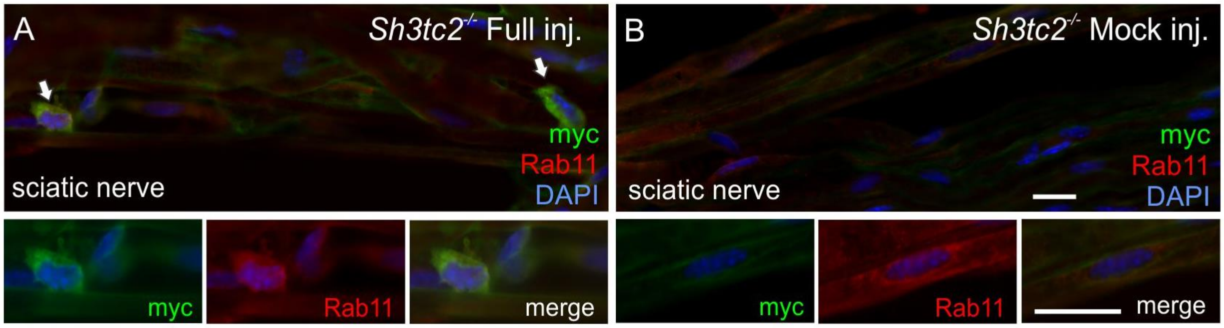

Supplementary Fig. 6: Virally expressed hSH3TC2 shows colocalization with its physiologically interacting protein Rab11. Double immunostaining of sciatic nerve teased fibers from LV-*Mpz.SH3TC2.myc*-injected (**A**) and untreated (**B**) *Sh3tc2*$^{-/-}$ mice shows expression of Rab11 in perinuclear Schwann cell cytoplasm (arrows) in both cases, where it shows colocalization with the myc-immunoreactive hSH3TC2 expressed in vector injected mice (arrows in **A**). Untreated mouse fibers show no myc immunoreactivity (**B**). Scale bars: 10 μm

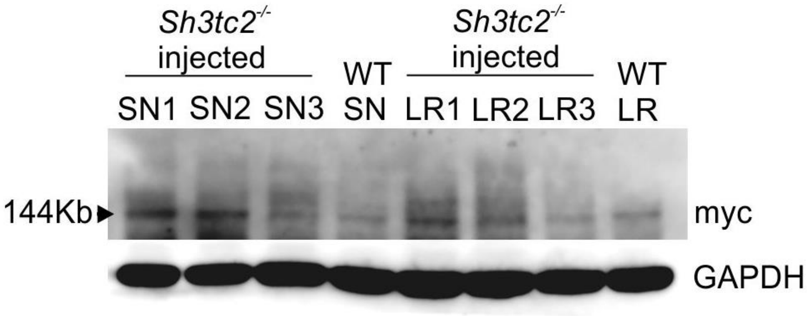

Supplementary Fig. 7: Immunoblot analysis of hSH3TC2 expression. Immunoblot of myc-tagged SH3TC2 protein in sciatic nerve (SN) and lumbar root (LR) samples of n=3 *Sh3tc2*$^{-/-}$ mice following intrathecal injection compared to untreated WT mouse (negative control). A specific band at the predicted size of hSH3TC2 (~144 kDa) is detected in two of the mice examined, while in the third one this does not differentiate from the background band in the negative control. GAPDH blot is shown under the myc blot for loading control.

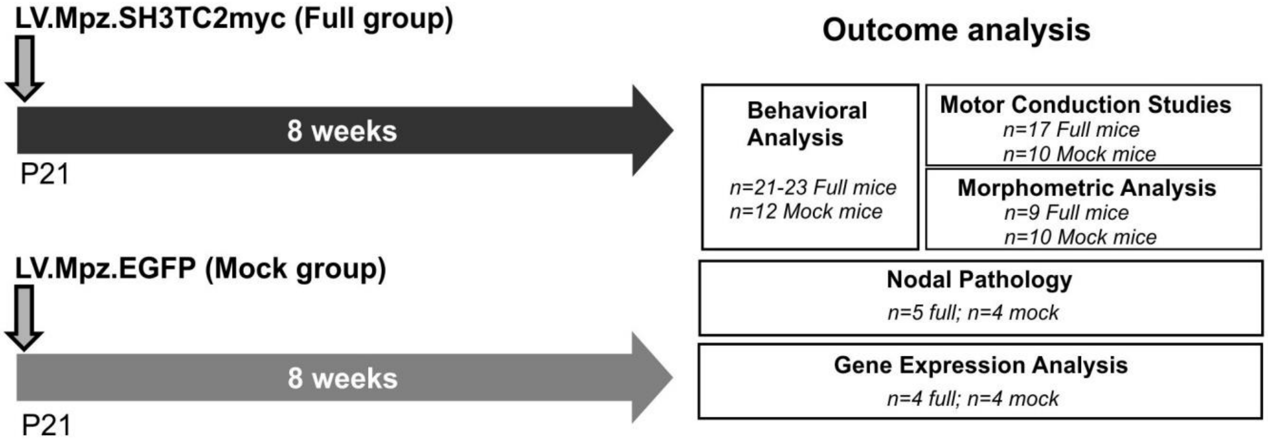

Supplementary Fig. 8: Diagram showing the study design for gene therapy in CMT4C mice. *Sh3tc2*$^{-/-}$ littermate mice were obtained from *Sh3tc2*$^{-/-}$ breeding pairs and used at P21 in order to perform intrathecal injections of either LV-*Mpz.SH3TC2.myc* (fully treated group) or LV-*Mpz.Egfp* (mock-treated group). Analysis was performed 8 weeks later at P77, with behavioral testing followed by pathological examination, or motor conduction velocities (details of numbers per group are provided in the methods and in each result shown). Additional groups of fully or mock-treated mice were used for immunostaining to assess nodal pathology and for RNA extraction for gene expression analysis.

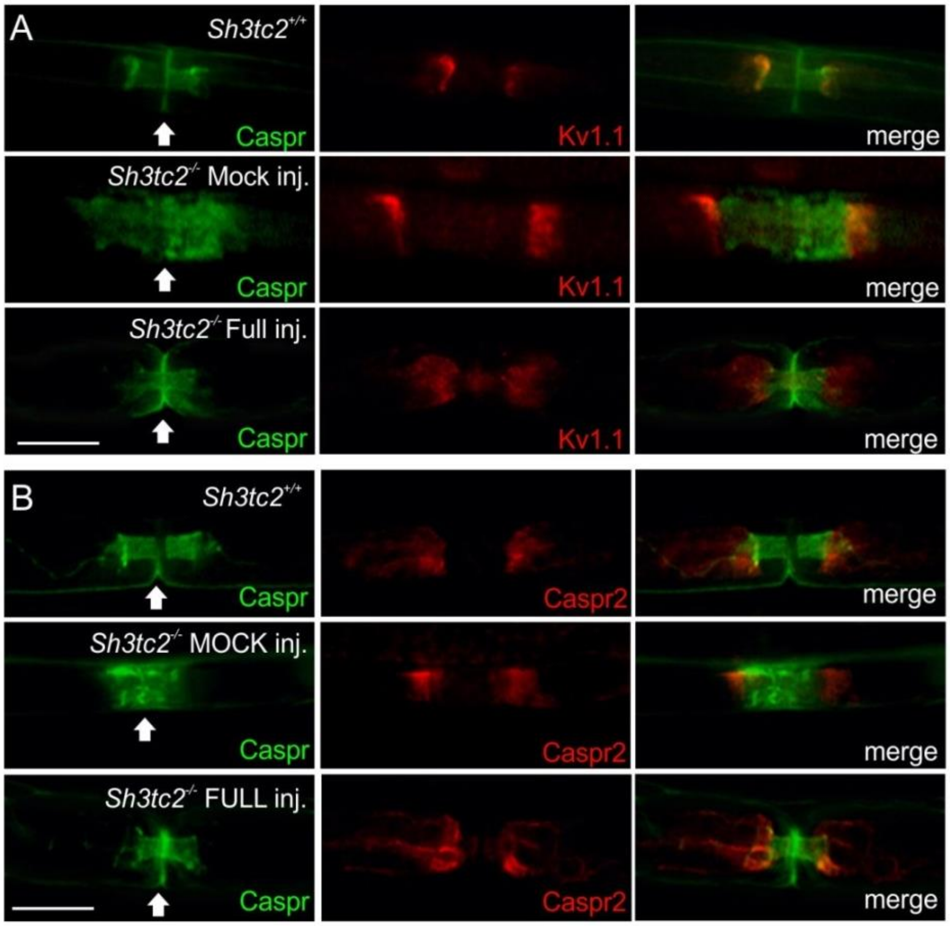

Supplementary Fig. 9: Improvement of nodal phenotype in treated *Sh3tc2*$^{-/-}$ mice. Immunostaining of sciatic nerve teased fibers from WT, mock-treated and fully treated *Sh3tc2*$^{-/-}$ mice with paranodal (Caspr, green) and juxtaparanodal (Kv1.1 or Caspr2, red) markers, as indicated, reveals the typical elongation of nodal width in mock-treated *Sh3tc2*$^{-/-}$ animals. This abnormality is improved in treated *Sh3tc2*$^{-/-}$ mice resembling more the appearance of WT nodal areas, consistent with improved nodal molecular architecture. Scale bar: 10μm

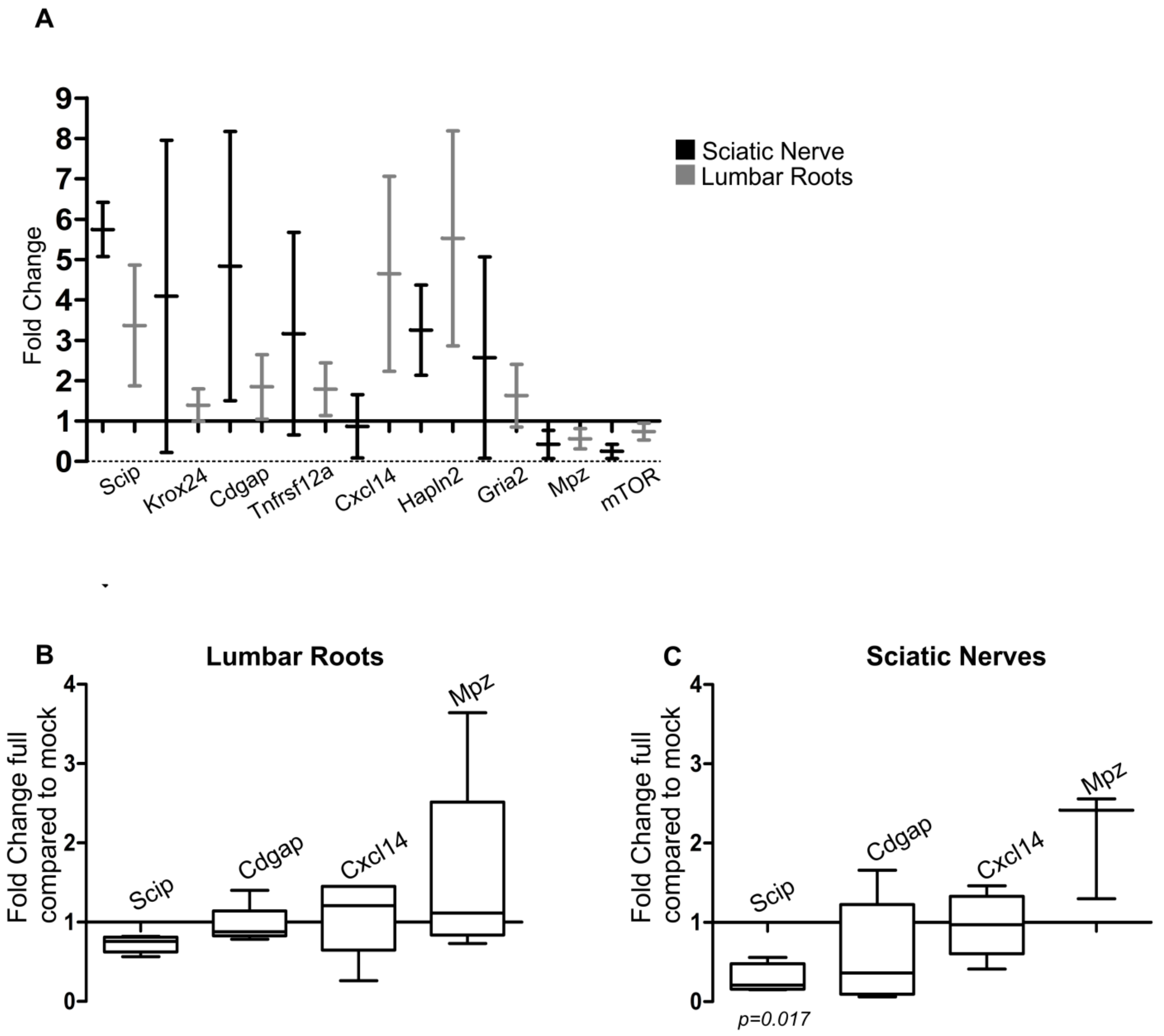

Supplementary Fig. 10: Gene expression analysis at baseline in 11-week old *Sh3tc2*$^{-/-}$ mice and in the treatment groups. A: The expression of several genes was compared by quantitative PCR between 11-week-old *Sh3tc2*$^{-/-}$ mice relative to WT littermates (here represented as baseline), confirming upregulation of several genes in *Sh3tc2*$^{-/-}$ mice, and mild downregulation of Mpz. These changes are more pronounced in the sciatic nerves and are found to a lesser degree in lumbar roots. Results of quantitative **B-C:** PCR analysis of the expression of selected genes in lumbar root (**B**) and in sciatic nerve (**C**) samples from groups of full vector treated compared to mock-treated *Sh3tc2*$^{-/-}$ mice at 11 weeks of age. Fold changes of gene expression in tissues from

full vector treated mice (n=4) relative to mock-treated mice (n=4) (considered as baseline) are shown. This analysis revealed a trend for reduced expression of genes that are upregulated at baseline in *Sh3tc2*$^{-/-}$ mice following treatment, including Scip, Cdgap and Cxcl14, as well as a slight reversal of Mpz downregulation. These changes were statistically significant only for the reduced Scip expression in sciatic nerves of treated mice (two-tailed Student’s t test).
